# Machine‐Learning Analysis of *Streptomyces coelicolor* Transcriptomes Reveals a Transcription Regulatory Network Encompassing Biosynthetic Gene Clusters

**DOI:** 10.1002/advs.202403912

**Published:** 2024-09-12

**Authors:** Yongjae Lee, Donghui Choe, Bernhard O. Palsson, Byung‐Kwan Cho

**Affiliations:** ^1^ Department of Biological Sciences Korea Advanced Institute of Science and Technology Daejeon 34141 Republic of Korea; ^2^ Department of Bioengineering University of California San Diego La Jolla CA 92093 USA; ^3^ Novo Nordisk Foundation Center for Biosustainability Technical University of Denmark Kemitorvet, Kongens Lyngby 2800 Denmark; ^4^ KI for the BioCentury Korea Advanced Institute of Science and Technology Daejeon 34141 Republic of Korea; ^5^ Graduate School of Engineering Biology Korea Advanced Institute of Science and Technology Daejeon 34141 Republic of Korea

**Keywords:** biosynthetic gene cluster, independent component analysis, machine learning, secondary metabolites, streptomyces, transcriptional regulatory network

## Abstract

*Streptomyces* produces diverse secondary metabolites of biopharmaceutical importance, yet the rate of biosynthesis of these metabolites is often hampered by complex transcriptional regulation. Therefore, a fundamental understanding of transcriptional regulation in *Streptomyces* is key to fully harness its genetic potential. Here, independent component analysis (ICA) of 454 high‐quality gene expression profiles of the model species *Streptomyces coelicolor* is performed, of which 249 profiles are newly generated for *S. coelicolor* cultivated on 20 different carbon sources and 64 engineered strains with overexpressed sigma factors. ICA of the transcriptome dataset reveals 117 independently modulated groups of genes (iModulons), which account for 81.6% of the variance in the dataset. The genes in each iModulon are involved in specific cellular responses, which are often transcriptionally controlled by specific regulators. Also, iModulons accurately predict 25 secondary metabolite biosynthetic gene clusters encoded in the genome. This systemic analysis leads to reveal the functions of previously uncharacterized genes, putative regulons for 40 transcriptional regulators, including 30 sigma factors, and regulation of secondary metabolism via phosphate‐ and iron‐dependent mechanisms in *S. coelicolor*. ICA of large transcriptomic datasets thus enlightens a new and fundamental understanding of transcriptional regulation of secondary metabolite synthesis along with interconnected metabolic processes in *Streptomyces*.

## Graphical abstract

*Streptomyces* produces pharmaceutically important secondary metabolites. To harness their genetic potential with understanding transcriptional regulation, independent component analysis is conducted on 454 transcriptomic profiles of *Streptomyces coelicolor*, revealing 117 independently modulated groups of genes (iModulons). iModulons can be utilized to predict the functions of uncharacterized genes and regulons of transcriptional regulators. iModulons provide fundamental insights toward secondary metabolism of *Streptomyces*.

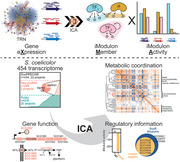

## 1 Introduction

*Streptomyces* represent a rich reservoir of secondary metabolites that has been extensively investigated to identify novel value‐added biochemicals, such as antibiotics, anthelmintics, and antitumor and immunosuppressive agents.^[^ ^1^ ^]^ In particular, the identification of numerous secondary metabolite biosynthetic gene clusters (smBGCs) for novel secondary metabolites has drawn great attention.^[^ ^2^ ^]^ Because of its non‐essential nature, secondary metabolism is tightly regulated and activated upon specific demands, limiting secondary metabolite production or causing complete smBGC inactivation under laboratory culture conditions.^[^ ^3^ ^]^ Various attempts have been made to overcome these limitations, including promoter refactoring of smBGCs,^[^ ^4^ ^]^ diversifying culture conditions,^[^ ^5^ ^]^ and heterologous expression of smBGCs,^[^ ^6^ ^]^ however none have proven consistently successful. The major obstacle in this context is the complex transcriptional regulation of smBGCs; for example, the pathway‐specific activator of the extensively studied actinorhodin biosynthetic gene cluster (BGC) is directly regulated by more than ten transcriptional regulators.^[^ ^7^ ^]^ Thus, a thorough understanding of transcriptional regulation in *Streptomyces* is crucial for increasing secondary metabolite production and activating silent smBGCs.

RNA sequencing (RNA‐seq) is an effective tool for investigating transcriptional regulation in microbes under various conditions.^[^ ^8^ ^]^ The use of independent component analysis (ICA) for multiple samples represents a recently introduced novel approach for analyzing transcriptome datasets.^[^ ^9^ ^]^ ICA is a machine‐learning algorithm developed to separate mixed signals into individual signals that are independent of each other.^[^ ^10^ ^]^ The genes located in the periphery of the transcriptional regulatory network, under the regulation of the most downstream regulator, are likely to be the target of just one or a few regulators in bacteria.^[^ ^11^ ^]^ Thus, the bacterial transcriptome dataset is an important target for applying ICA to dissect transcriptional regulation. ICA of large transcriptomic datasets can simplify the data structure for easy analysis by grouping thousands of genes into a much smaller number of independently modulated groups, called iModulons, based on their expression patterns across the experimental condition.^[^ ^9^ ^]^ The transcriptional coordination of genes in each iModulon may represent their functional and/or regulatory associations, thus expanding our understanding of gene functions and their transcriptional regulation.

*Streptomyces* possesses twice as many genes and double the number of transcriptional regulators compared to other bacteria.^[^ ^12^ ^]^ Thus, dimensionality reduction, which involves grouping thousands of genes into smaller numbers of iModulons, by ICA can revolutionize the current transcriptomic analysis of *Streptomyces*. The only requirement for applying ICA is the availability of a sufficient number of high‐quality transcriptome datasets.^[^ ^9^ ^]^ Here, to expand our knowledge of the regulation of secondary metabolism in *Streptomyces*, we applied ICA to *S. coelicolor*, a model *Streptomyces* species that has possibly undergone the most RNA‐seq experiments among *Streptomyces*. Using this approach, we identified the iModulons associated with 25 smBGCs. We also generated an RNA‐seq dataset of sigma factor overexpressing strains to analyze their potential role in antibiotic regulation.

## 2 Results

### 2.1 *Streptomyces coelicolor* Transcriptome Contains 117 Independently Regulated Sets of Genes

We initiated this study by aggregating publicly available transcriptomic data from 205 samples across 20 distinct projects (Table S1, Supporting Information). The inherent challenges in deciphering meaningful independently modulated signals within this dataset became apparent, primarily because of the recurrent utilization of the same media and a constrained range of experimental conditions, as underscored by the presence of over 50 time‐resolved samples from a single project. We addressed these limitations by generating RNA‐seq profiles of *S. coelicolor* M145 cultivated on 20 different carbon sources and 64 engineered strains, each with an overexpressed sigma factor. In particular, since *Streptomyces coelicolor* encodes 64 sigma factors in its genome,^[^ ^13^ ^]^ far more than the sigma factors of other bacteria,^[^ ^14^ ^]^ it is expected to use sigma factor‐dependent regulation for a significant portion of its transcriptional dynamics. Therefore, we overexpressed the sigma factors of *S. coelicolor* to induce transcriptomic perturbation which enabled us to detect their regulatory modalities (Figure S1 and Table S2, Supporting Information).

**Figure 1:**
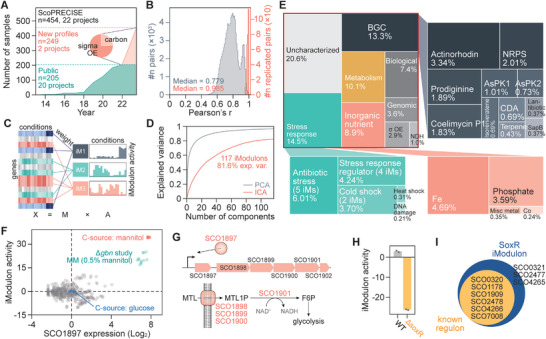
Independent component analysis (ICA) decomposes transcriptional regulatory signals from an expanded transcriptomic dataset of *Streptomyces coelicolor*. A) Composition of the publicly available dataset and the single‐carbon or sigma factor‐perturbation experiments. B) Pairwise Pearson correlation between transcriptomic samples, comparing replicates with randomly selected pairs of transcriptomes. C) Gene expression data (**X** matrix, genes are rows and columns are conditions) is decomposed into independently regulated genetic modules (**M**, weightings in columns determine gene membership of iModulons) and the condition dependent activity of the iModulons (**A**, rows are iModulons, columns are their activities in a given condition). D) ICA analysis reveals 117 robust iModulons explaining 81.6% of the variance in the dataset. E) A breakdown of the combined explained variances of iModulons by their category. BGC, biosynthetic gene cluster. OE, overexpression. NDH, NADH dehydrogenase. F) The MtlR iModulon activity plot indicates the involvement of the iModulon in mannitol utilization. G) The MtlR iModulon is composed of a transcription factor, sugar transporter, and sugar alcohol dehydrogenase. H) Activity of the SoxR iModulon in the deletion mutant strain. Dots indicate individual samples. I) Members of the SoxR iModulon match closely with those of the known SoxR regulon.

The high‐quality consolidated dataset (from literature plus generated in this study) consisted of 454 profiles from 22 projects with a median replicate correlation of 0.99 (**Figure** **1**A,B). This dataset was subjected to ICA. Sets of independently co‐regulated genes were identified from the observed gene expression levels across diverse conditions (Figure 1C). Note that only samples with extremely high correlation between biological replicates (Pearson's r > 0.95) were used to reduce false negative genes in the decomposed components, arising from the blurred expressional integrity in poor quality samples (Figure 1B). Based on the ICA decomposition, we obtained 117 robust iModulons, which explained 81.6% of the variance in observed gene expression (Figure 1D; Tables S3–S7, Supporting Information). Based on the genes constituting the iModulons, the iModulons were functionally annotated (Table S8, Supporting Information). iModulons composed of genes of unknown function accounted for 20.6% of the variance in expression (Figure 1E). This high percentage was not surprising since a large portion of *S. coelicolor* genes are functionally unannotated, highlighting our incomplete understanding of this industrially important bacterium. Interestingly, the iModulons majorly consisting of smBGCs predicted by AntiSMASH (v7.1.0) accounted for 13.3% of the total expression variance in the dataset (Figure 1E).^[^ ^15^ ^]^ Given the importance of this strain for the antibiotic‐production industry, this finding is particularly interesting. The remaining transcriptional variation was explained by iModulons for stress response, metabolism, and inorganic nutrient acquisition.

Synchronized expression is advantageous for a group of genes with similar functions; thus, ICA may help identify a group of genes required for a specific metabolism.^[^ ^9^ ^]^ In particular, the expanded experimental conditions for different carbon source supplies allowed for the identification of metabolic pathways, since the iModulons responsible for specific substrate utilization were distinctively activated when the substrate was used as the sole carbon source (Figure S2, Supporting Information). For example, an iModulon composed of six genes encoding a putative transcription factor (SCO1897; similar to *Escherichia coli* galactitol utilization operon repressor; 33.8% identity, BLAST), putative sugar transporter components (SCO1898‐1900; similar to sorbitol/mannitol transporter from *Rhodobacter sphaeroides*; 40.6–42.8%), and a predicted zinc‐binding dehydrogenase (SCO1901) sharing similarity with sorbitol dehydrogenase from *Bacillus subtilis* (30.1% identity; BLAST) was active in the presence of mannitol, indicating its involvement in mannitol utilization (Figure 1F; Figure S2, Supporting Information). Thus, the sugar alcohol transporters and dehydrogenase encoded by this iModulon can be reasonably assumed to be responsible for mannitol utilization (Figure 1G).

In addition to functional relevance in the iModulon‐captured genes, the transcriptional coordination of the members in an iModulon may indicate the regulation by a specific transcriptional regulator.^[^ ^9^, ^16^ ^]^ To assess the robustness of iModulons for capturing transcriptional regulation, we compiled experimentally validated regulatory information of the bacterium, and compared the known regulons with those reconstituted by iModulons (Table S9, Supporting Information).^[^ ^17^ ^]^ Many iModulon genes exhibited considerable similarities to known regulons in terms of gene membership, proving the robustness of iModulons for capturing transcriptional regulation, and at the same time, the biological significance and accuracy for the ICA‐based transcriptome analysis (Figure S3, Supporting Information). For one instance, one iModulon showed an extreme activity reduction in a knockout experiment with *soxR*, which encodes an actinorhodin‐sensitive transcriptional regulator,^[^ ^18^ ^]^ in contrast to the control strain (Figure 1H), indicating regulation of the iModulon by SoxR. This iModulon consisted of nine genes, six of which were previously known as SoxR regulon members (Figure 1I).^[^ ^17b^ ^]^ The remaining three genes were neighbors of the SoxR regulon, potentially indicating a larger SoxR regulon or transcriptional read‐through. Thus, iModulon information delivers broader regulatory information compared to protein‐DNA interaction assays. Collectively, the iModulons identified by ICA shed light on the functions of previously unknown genes and their transcriptional regulatory networks.

### 2.2 ICA Elucidates a Global Transcriptional Regulatory Network in *S. coelicolor*

**Figure 2:**
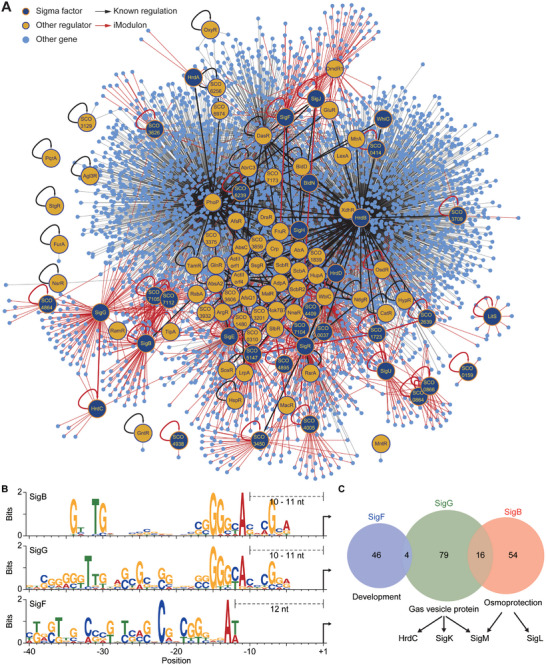
The transcriptional regulatory network of *Streptomyces coelicolor* reconstructed from iModulons. A) The transcriptional regulatory network of *S. coelicolor* reconstructed by combining known transcriptional regulation with the regulatory iModulons identified in this study. Red arrows indicate iModulon membership of the responsible regulator and black arrows indicate previously known regulations. The circles indicate genes involved in the transcriptional regulatory network. Sigma factors and transcription factors with known targets or assigned iModulons are colored navy and yellow, respectively. B) Conserved promoter motif identified in the genes of the iModulons related to the SigB homologs. Arrows indicate the transcription start sites. C) Venn diagram of the gene membership of the three iModulons related to SigB homologs. Arrows indicate target sigma factors hypothesized to be regulated by the SigB homologs, as revealed by iModulon gene membership.

Owing to the ability of ICA to capture the regulons of transcriptional regulators, we could assign the putative regulators for 39 iModulons based on known regulatory information, enrichment of putative regulator binding motifs, and/or specific iModulon activity in transcriptionally perturbed samples (Table S10, Supporting Information). In particular, the RNA‐seq dataset from sigma factor‐overexpressing samples resulted in the identification of iModulons that were activated solely in those strains (*n* = 19) (Figure S4, Supporting Information). Among the identified regulatory iModulons, 28 were associated with 30 sigma factors, which accounted for approximately half of the sigma factors of *S. coelicolor* (64 sigma factors are found on the chromosome).^[^ ^12^, ^13^ ^]^ ICA revealed that more than half of the characterized sigma factors regulated a few genomic loci (<5), indicating their involvement in specialized functions at the periphery of the transcriptional regulatory network (Table S7, Supporting Information). By combining previously known regulation data (Table S9, Supporting Information) with the regulation data inferred from iModulons,^[^ ^17^ ^]^ we reconstructed the global transcriptional regulatory network of *S. coelicolor* (**Figure** **2**A). The reconstructed network consisted of 4396 genes, including 100 regulators and 6280 interactions, and iModulons provided information on 29 regulators and 958 interactions that were not previously characterized. The putative regulatory information from ICA was especially important for understanding the transcriptional regulation of *S. coelicolor*, since the iModulon information expanded 70% of the previously known interactions when excluding the 3955 interactions governed by only two regulators, PhoP and HrdB (Table S9, Supporting Information). As shown in Figure 2A, the regulators are enriched in the vicinity of ActII‐orf4, suggesting that the current knowledge about the transcriptional regulation of *S. coelicolor* is highly biased toward secondary metabolism. Thus, the unbiased regulation information inferred from iModulons will serve as a valuable resource for understanding the transcriptional regulation of *S. coelicolor*.

On the other hand, we also observed putative sigma factor iModulons with more global regulation, and to our surprise, similar promoter motifs were detected in multiple sigma factor iModulons associated with the SigE/SigR and SigB homologs (Figure 2B; Figure S5A, Supporting Information). These sigma factors are associated with stress responses, specializing in cell envelope stress,^[^ ^19^ ^]^ thiol‐oxidative stress,^[^ ^20^ ^]^ and osmotic stress.^[^ ^21^ ^]^ Interestingly, the stress‐response iModulons shared few members despite the high similarity of their respective promoter motifs (Figure S5B, Supporting Information). Only the SigB homolog iModulons, SigB iModulon, and SigG iModulon shared a relatively high number of gene members, and they seemed to co‐regulate SigM, another SigB homolog (Figure 2C). The presence of a common promoter motif in sigma factor homologs may suggest that these sigma factors diverged from a common ancestral gene involved in a broad range of stress responses into individual factors involved in specialized stress responses. In addition, these regulatory elements are often conserved across streptomycetes. Comparison of the promoter sequences of the 15 regulatory iModulons with potential regulator binding sites (detected by MEME suite,^[^ ^22^ ^]^ Table S10, Supporting Information) with those of homologous genes (*E*‐value < 10^−10^ from protein BLAST) in other *Streptomyces* species, including *S. avermitilis*, *S. clavuligerus*, *S. griseus*, and *S. lividans*,^[^ ^23^ ^]^ revealed a significant conservation of regulatory motifs (Figure S6, Supporting Information). This suggests that iModulon information in *S. coelicolor* may help elucidate transcriptional regulations in other *Streptomyces* species, further expanding its value.

### 2.3 iModulons Could Accurately Predict 25 smBGCs

**Figure 3:**
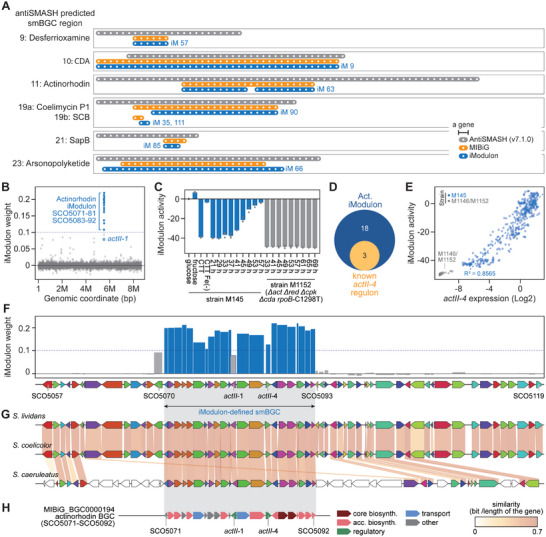
Detection of the actinorhodin biosynthetic gene cluster using iModulons. A) Genetic constituents of BGCs detected by iModulon agree with BGCs in MIBiG that are complete (BGCs encoding all the necessary components for producing the compound). Each dot represents a gene. Gray, orange, iModulon strips are BGCs predicted by AntiSMASH, MIBiG, and iModulon, respectively. CDA: calcium‐dependent antibiotic. SCB: *S. coelicolor* butanolides. B) Weights of genes in the actinorhodin iModulon and its genomic location. Genes (blue) with high weights are members of the actinorhodin iModulon. The dashed line represents the cutoff for iModulon gene membership. C) Actinorhodin iModulon activity under diverse conditions and WT and genome edited strains. Experimental conditions included different carbon sources (substrates), iron limitation, and time‐series sampling. Strain M1152 is a BGC‐free derivative of strain M145. Dots are individual replicates. D) Comparison between gene membership of the actinorhodin iModulon and the known ActII‐orf4 regulon. E) Correlation between the activities of the actinorhodin iModulon and actII‐orf4 expression. Dots are individual transcriptomic samples. F) Schematic representation of the genomic locus for actinorhodin biosynthesis predicted using antiSMASH (v7.1.0). The genes in the iModulon are shown and the bars show the iModulon weights for each gene. G) Alignment of *S. coelicolor* actinorhodin BGC to that of different *Streptomyces* species. The shades indicate sequence conservation. The similarity score is defined as the bit score (BLAST) divided by the length of the gene. H) Actinorhodin BGC annotated by MIBiG database. Genes are colored by their functional category. biosynth: biosynthetic gene, acc. biosynth: accessory biosynthetic genes.

Actinomycetes produce numerous secondary metabolites.^[^ ^1^ ^]^ However, identification of the genes responsible for the production of these secondary metabolites requires extensive experimental effort.^[^ ^24^ ^]^ Considering the proven robustness of ICA in capturing individually modulated genes, we aimed to identify the iModulons associated with secondary metabolite biosynthesis, hypothesizing that biosynthetic genes are likely to be modulated together. The most widely used computational tool for smBGC prediction, AntiSMASH (v7.1.0),^[^ ^15^ ^]^ predicted 31 smBGCs in the 27 regions of *S. coelicolor* genome (note that one BGC region could have multiple BGCs). 21 potential smBGCs were identified using the well‐recognized and curated BGC database MIBiG (v3.0).^[^ ^25^ ^]^ By utilizing the in silico inferred smBGC information, iModulons were searched for the presence of secondary metabolite biosynthetic genes, and the smBGCs could be redefined based on the iModulon members neighboring the biosynthetic genes. Overall, ICA redefined the BGC boundaries of 25 out of 31 antiSMASH‐detected BGCs (**Figure** **3**A; Figure S7, Supporting Information), in agreement with the 21 experimentally validated BGCs from the MIBiG database.

We inspected the biosynthetic iModulon for actinorhodin, the most representative secondary metabolite produced by *S. coelicolor*. This iModulon consisted of 21 genes (Figure 3B). The activity of the iModulon progressively increased as growth moved toward the stationary phase, consistent with previous observations,^[^ ^8^, ^26^ ^]^ and was silenced in the BGC‐free strain (Figure 3C).^[^ ^27^ ^]^ The iModulon included 18 additional genes compared to the dedicated actinorhodin regulator ActII‐orf4 (Figure 3D),^[^ ^28^ ^]^ which is highly likely to include the operonic structure, and the activity of the iModulon correlated with that of ActII‐orf4 (Figure 3E), suggesting that ActII‐orf4 is a potential regulator of this iModulon and emphasizing the ability of ICA to capture broader regulons compared to transcription factor binding assays. However, the iModulon overlapped with multiple regulators (Figure S8, Supporting Information),^[^ ^17a^ ^]^ indicating complex regulatory signals governing actinorhodin production. Importantly, it covered only a fraction of the antiSMASH‐predicted clusters (Figure 3F). When compared to the actinorhodin clusters of related species, many genes were not conserved in distal relatives (Figure 3G). AntiSMASH uses a conservative cutoff to ensure that genes are not lost, and extra genes are frequently observed. The conserved cluster matched the iModulon and perfectly matched the cluster annotated by the MIBiG dataset, an experimentally and widely curated database (Figure 3H).

Furthermore, when the completed clusters (*n* = 6) in which the predicted genes were sufficient to support the production of the compound by MIBiG were compared with the iModulons, they matched almost perfectly (Figure 3A). In addition, there are numerous experimental evidences supporting the accuracy of iModulon refined smBGC regions for 12 secondary metabolites (Table S11, Supporting Information).^[^ ^29^ ^]^ Especially, disruption of the genes inside actinorhodin BGC iModulon at both of the left and right boundaries resulted in decreased blue pigment production, while the disruption of the gene outside of the left boundary showed no effect for actinorhodin production, indicating accurate prediction of smBGC boundary using iModulon.^[^ ^29b^ ^]^ Notably, undecylprodigiosin captured not only the BGC, but also the EcrA1/A2 two‐component system (TCS) and a putative membrane protein (SCO2519) (Figure S7, Supporting Information).^[^ ^30^ ^]^ According to a previous report, the TCS positively regulates undecylprodigiosin production.^[^ ^30^ ^]^ Additionally, SCO2519 is a paralog of the membrane protein ActII‐ORF3 with a putative antibiotic efflux function. Since the production of these secondary metabolites with antibiotic activity is often accompanied by a dedicated exporter,^[^ ^31^ ^]^ SCO2519 may be an exporter for undecylprodigiosin, which is required for self‐resistance. This highlights the robustness and accuracy of iModulon based smBGC refinement.

### 2.4 Iron–Phosphate Axis in Actinorhodin Production

**Figure 4:**
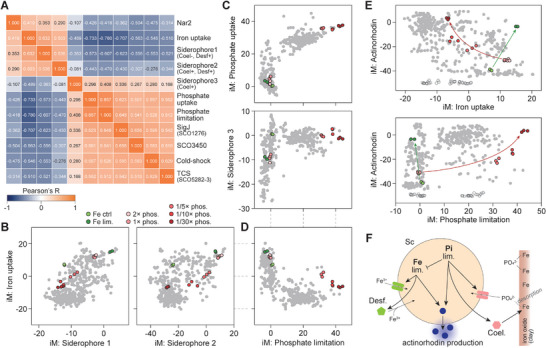
Iron–phosphate stress affects actinorhodin production. A) Stress iModulons clustered in the vicinity of the biosynthetic iModulons based on activity correlation. B) Activity relationship between iron‐uptake iModulons and siderophore iModulons. Gray dots are all conditions in the data set. Highlighted conditions are colored as the legend indicates. C) Activity relationship between phosphate‐limitation iModulons and phosphate‐uptake and siderophore iModulons. D) Activity relationship between iron‐uptake iModulons and phosphate‐limitation iModulons. E) Both iron limitation and phosphate limitation promote actinorhodin production. F) Schematic diagram representing the regulation of actinorhodin production by iron and phosphate stress.

As shown in the actinorhodin BGCs, multiple regulatory mechanisms may mediate precise control of secondary metabolism. Since secondary metabolites function as signaling molecules in various physiological processes in this bacterium, such as morphogenesis, development, and stress response,^[^ ^32^ ^]^ we further investigated the iModulons associated with smBGCs. Activity correlations between the iModulons across experimental conditions were examined using hierarchical clustering (Figure S9A, Supporting Information). Synchronized co‐expression of the secondary metabolite iModulons, including those for calcium‐dependent antibiotic, coelimycin, undecylprodigiosin, and actinorhodin, and a few metabolic iModulons (purines and fatty acids) may provide precursors to the biosynthetic pathways. We specifically focused on the stress‐response iModulons clustered next to the biosynthetic iModulons. This cluster consisted of several iModulons associated with stress responses, such as the hypoxia/anoxia‐responsive nitrate assimilation system (Nar2),^[^ ^33^ ^]^ cold‐shock proteins, iron acquisition, and phosphate acquisition (**Figure** **4**A; Figure S9B, Supporting Information). Since iron‐ and phosphate‐associated iModulons contributed to >8% of the transcriptional variation (Figure 1E), we hypothesized that the iron–phosphate regulatory axis plays a considerable role in transcriptomic changes in *S. coelicolor*.

To address this hypothesis, we generated additional transcriptomic samples with different phosphate concentrations ranging from 2‐fold excess to 1/30 of the normal condition (Tables S12 and S13, Supporting Information). Four iModulons related to iron acquisition were identified, of which one (“iron uptake”) encoded iron transporters, and the others encoded two iron‐siderophores with different regulatory regimens (Figure 4A). The iron uptake, siderophore 1, and siderophore 2 iModulons were activated by iron limitation (induced by the addition of the iron‐chelator, 2,2′‐bipyridyl to the culture medium) (Figure 4B). On the other hand, two phosphate‐responsive iModulons, “phosphate uptake” and “phosphate limitation,” encoding phosphate transporters and phosphatases, respectively, were activated under phosphate‐limited conditions. However, phosphatases showed delayed expression, indicating that the cells decomposed their own materials, when necessary, only after seeking external phosphates (Figure 4C). Interestingly, the third siderophore iModulon (“siderophore 3”) encoding coelichelin was activated by phosphate limitation rather than iron limitation (Figure 4C). This phenomenon is similar to that observed in marine bacteria and pathogens, since they dissolve Fe‐containing minerals (such as iron oxide) to access adsorbed phosphates and during pathogenesis.^[^ ^34^ ^]^ Thus, *S. coelicolor* has two different siderophores with different physiological roles: one for iron uptake and competition in the bacterial community and the other for phosphate stress.^[^ ^26^ ^]^

Furthermore, the iron and phosphate stress iModulons exhibited an inverse correlation (Figure 4D). Specifically, when cells were exposed to phosphate‐limiting conditions, iron uptake was reduced (Figure 4B,D), whereas iron limitation did not affect phosphate iModulon activity (Figure 4C,D). This finding indicates the presence of a regulatory scheme for the iron–phosphate axis, suggesting that cells prioritize phosphate over iron. Since iron limitation can trigger actinorhodin production,^[^ ^26^ ^]^ this may indicate a potential association between phosphate limitation and antibiotic production. Close inspection revealed that both stresses could activate the actinorhodin iModulon (Figure 4E). The activation of the actinorhodin iModulon by phosphate stress represents a distinct mechanism in comparison with the response induced by iron limitation, since phosphate‐limitation conditions repress the iron‐acquisition response. To further investigate the involvement of other metabolisms in iron‐phosphate dependent actinorhodin regulation, the activities of metabolism related iModulons were compared with the activities of actinorhodin iModulon across the conditions. Overall, 7 metabolism related iModulons moderately coordinated with the actinorhodin iModulon (|Pearson's r| > 0.5, Figure S10A, Supporting Information). Their activities were further compared with the iron uptake iModulon and phosphate limitation iModulon. Interestingly, iModulons related to carbon metabolisms and respiration coordinated with both iron uptake and phosphate limitation iModulons, while nitrogen and fatty acid related iModulons did not (Figure S10B,C, Supporting Information). This suggests the presence of another layer of regulation by nitrogen and fatty acid, as well as interconnected regulation of carbon metabolism and respiration with iron‐phosphate axis. On the other hand, the iron‐phosphate axis were further investigated for the regulatory role in other secondary metabolism. Phosphate stress also weakly induced a few smBGC iModulons (Figure S11A, Supporting Information), while most of the iModulons involved in secondary metabolites were related to neither phosphate nor iron stresses (Figures S11 and S12, Supporting Information). Therefore, the ICA revealed a new link between antibiotic production and phosphate limitation in *S. coelicolor* (Figure 4F).

### 2.5 Transcriptional Perturbation Through Sigma Factor Overexpression Affects Secondary Metabolite Production

**Figure 5:**
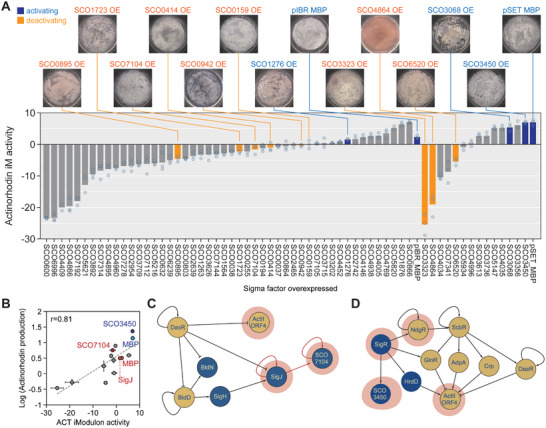
Perturbation of the transcriptional regulatory network affects actinorhodin production. A) Activity of the actinorhodin iModulon across the sigma factor overexpression conditions (Table S6, Supporting Information). Photographs of the overexpressing strains in R5−solid media were taken at the RNA‐seq sampling time points. Dots indicate the individual transcriptomic samples. B) iModulon activity reflects the actual actinorhodin production level. R is the Pearson's correlation coefficient. Individual dots indicate the average value of the biological triplicates, and error bars represent the standard deviation. C,D) The reconstructed regulatory cascade in the vicinity of (C) SigJ, (D) SCO3450, and ActII‐orf4. Transcription factors and sigma factors are colored in yellow and blue, respectively. Red shade indicates iModulon‐assigned regulator and red arrow indicates probable regulation inferred from iModulon membership.

The biosynthesis of actinorhodin was also investigated to determine whether antibiotic production in *S. coelicolor* could be affected by sigma factors. Differing levels of actinorhodin iModulon activity were observed along with overexpression of sigma factors, suggesting differential effects of sigma factors on actinorhodin production (**Figure** **5**A). In general, overexpression of sigma factors appeared to inhibit actinorhodin production due to the higher activity of the actinorhodin iModulon in control strains. The function of overexpressed sigma factors may induce inhibitory effects on actinorhodin production. On the other hand, cellular burden from transcriptomic perturbation could be another reason for the inhibition of actinorhodin production, considering the failure to express some sigma factors and chromosomal deletion in three strains each expressing maltose‐binding protein (MBP)‐fused SigB, SigN, and SCO3709 (Table S2, Supporting Information). Although the type of plasmid used for sigma factor overexpression seemed to affect actinorhodin production, the actinorhodin iModulon activity was highly correlated with the actual production level (Figure 5B; Pearson's R = 0.81).

As described in the previous section, two iModulons regulated by SCO3450 and SigJ were clustered near the phosphate–iron stress responder iModulons, suggesting the involvement of these two sigma factors in phosphate regulation and actinorhodin production (Figure 5A; Figure S9B, Supporting Information). As expected, both iModulons were activated under phosphate‐limiting conditions, and the activities of the two iModulons weakly correlated with the activity of the actinorhodin iModulon (Figure S13, Supporting Information; Pearson's R = 0.51 and 0.49, respectively). Although evidence for the direct regulation of the actinorhodin BGC by SigJ and SCO3450 has not been found in the literature, the regulatory network indicates that their upstream regulators are involved in the regulation of the actinorhodin BGC (Figure 5C,D). However, the strain overexpressing SigR, the upstream regulator of SCO3450, showed lower activity of the actinorhodin iModulon in comparison with the strain overexpressing SCO3450, suggesting that activation is facilitated not by hierarchical regulation from the upstream sigma factors, or that inhibitory mechanisms may counteract the activation (Figure 5A). In contrast, SigJ potentially regulates SCO7104 on the basis of the iModulon structure, and SCO7104 appears to activate the actinorhodin BGC (Figure 5A). Interestingly, SigJ and SCO3450 seem to regulate catabolic genes clustered at specific genomic loci (SCO1265 – SCO1288 and SCO3437 – SCO3465, respectively), and their overexpression may promote phosphate consumption, resulting in actinorhodin production.

Overall, each sigma factor affects antibiotic production differently, and ICA can be used to detect the associated metabolic and transcriptional regulation of sigma factors. Our data provide insights into the modulation of secondary metabolite production and the regulation of secondary metabolism in *Streptomyces*.

## 3 Conclusion

Understanding regulation of secondary metabolism in *Streptomyces* has long been a topic of interest to enable identification of novel bioactive compounds and to improve antibiotic productivity. Here, with an RNA‐seq compendium composed of 454 samples, we used ICA to explore the transcriptional regulation in *S. coelicolor*, with a focus on secondary metabolism. ICA decomposed the transcriptomic dataset into 117 iModulons, 11 of which accounted for 13.3% of the transcriptomic variance. We showed that each iModulon consists of functionally relevant and transcriptionally coordinated genes, which revealed the cellular functions unknown genes are involved with, and the structure of the transcriptional regulatory network of *S. coelicolor*. BGC iModulons can elucidate secondary metabolism by accurately identifying sets of genes involved in the synthesis of secondary metabolites, an improvement over widely used computational tools. Moreover, the findings that BGC iModulons are coordinated with other iModulons can provide deep insights into the regulation of BGCs.

Our in‐house generated RNA‐seq dataset was obtained from *S. coelicolor* cultivated in 20 different carbon sources, revealing the genes required for utilization of each carbon source. Because publicly available RNA‐seq datasets were generated from complex media, our use of minimal media helped define the genes required for metabolism of specific substrates. In addition, by generating an RNA‐seq dataset from sigma factor‐overexpressing strains, putative regulons of approximately half of the sigma factors in *S. coelicolor* could be inferred using ICA. Although sigma factors were previously shown to be involved in specific functions, this legacy information included indirect and subsidiary effects or was often limited to a few target genes.^[^ ^21^, ^35^ ^]^ In contrast, ICA results provided novel and comprehensive insights into the regulation of 24 sigma factors, including functional annotations. When comparing iModulon membership with known regulatory information, there may present some discrepancies between the two groups, possibly arising from either inaccurate calling of iModulon members in each decomposed component or insufficient decomposition of a component from low diversity in RNA‐seq conditions (Figure S3, Supporting Information). Thus, iterative cycles of ICA‐based regulon prediction and experimental validation would aid elucidation of highly precise transcriptional regulatory network in *S. coelicolor*.

Because BGCs are often regulated by a dedicated pathway‐specific regulator, ICA aids to refine the gene content of smBGCs predicted from in silico tools such as antiSMASH and provides insights on the transcriptional regulation of the smBGCs.^[^ ^15^ ^]^ We were able to refine the genomic boundaries of 25 BGCs more precisely than the regions predicted by antiSMASH. Especially, iModulon‐based refinement of smBGCs may serve as an effective strategy for natural product discovery. Inherent challenges in novel secondary metabolite discovery include detection of the produced molecule, and to overcome the limitation, heterologous expression of the smBGCs in genetically engineered hosts with metabolically clean background is often conducted.^[^ ^36^ ^]^ As demonstrated in previous work, engrafting the whole iModulon members confers more optimal function,^[^ ^37^ ^]^ we believe that iModulon will provide valuable information for engineering smBGC pathways in heterologously, possibly in suitable *Streptomyces* host species. In addition, iModulon information can be utilized for improving secondary metabolite production. Currently, the routinely utilized complex medium lacks intellectual basis for production of desired secondary metabolites. A recent study describes how iModulon‐inferred relationship between two media components can guide altering media composition for optimal growth.^[^ ^38^ ^]^ Given that smBGC iModulon activity correlates with the actual production level (Figure 5B), the factors affecting secondary metabolite production, including carbon sources and phosphate concentration, can be elucidated from the iModulon activity in diverse RNA‐seq conditions, providing insights for optimizing culture medium. Furthermore, the coordinated activation of BGC iModulons with few metabolic iModulons indicates the importance of a timely supply of precursors for secondary metabolite production, which has been experimentally demonstrated.^[^ ^39^ ^]^ The degradation of triacylglycerols was correlated with the polyketide production, and controlling the expression of SCO6196, responsible for degradation of triacylglycerols evidenced by transcriptomic analysis, to coordinate with the expression of polyketide smBGCs resulted in higher production of polyketides. Especially, SCO6196 is the member of iModulon 62, related to CoA metabolism, and the iModulon carries 74 genes as the member. Controlling the expression of other members along with SCO6196 would be beneficial for high titer production of polyketides.^[^ ^37^, ^38^ ^]^

On the other hand, the activity of the actinorhodin iModulon is coordinated with multiple iModulons, including phosphate limitation and iron uptake, indicating complex regulation by multiple triggers. Among the phosphate limitation‐associated iModulons, two sigma factor iModulons, SCO3450 and SigJ, also coordinated with the actinorhodin iModulon, indicating their involvement in BGC regulation in a phosphate‐dependent manner. ICA thus not only identifies BGC iModulons, but also the house‐keeping iModulons involved in biosynthetic functions, as well. Thus, a global view of BGC regulation with metabolic interconnection has been revealed, and iModulon provides insights on future directions to further investigation the regulatory mechanisms of secondary metabolite production, such as identifying the potential regulators for iron‐phosphate dependent smBGC regulation.

Our study provides new and comprehensive information about the functions of sigma factors. Interestingly, SigJ is known to be regulated by SigH, a SigB homolog involved in development and the osmotic stress response.^[^ ^17d^ ^]^ While the SigB homologs captured as the iModulon shared a similar promoter motif, the overexpression of each homolog resulted in different levels of actinorhodin iModulon activity. Although overexpression of SigB homologs seemed to be unfavored by *S. coelicolor* (chromosomal deletion in SigB and SigN overexpression samples, failed expression of SigH), only two homologs, SigI and SigF, seemed to be favored for actinorhodin production. Thus, further investigation of the role of the SigB family of sigma factors in the regulation of secondary metabolism may be worthwhile.

In summary, we applied ICA to a large transcriptome dataset of *S. coelicolor* and investigated the transcriptional regulation of sigma factors of secondary metabolism. The iModulon information generated by ICA provides a valuable resource for deciphering the complex regulation of secondary metabolism in *Streptomyces*.

## 4 Experimental Section

### Strains and Culture Condition

For cultivation in 20 different carbon sources and different phosphate concentration, 1 × 10^8^ *S. coelicolor* M145 spores were inoculated in 50 mL of defined media in a 250‐mL baffled flask with 8 g of glass beads (3 ± 0.3 diameter) at 30 °C and 200 rpm. The defined media consisted of NH_4_Cl, Na_2_SO_4_, K_2_HPO_4_, MgCl_2_, MOPS, and each carbon source; the concentration of each component is listed in Table S14 (Supporting Information). After inoculation, the cells were transferred into fresh medium in triplicate, and the sampling conditions are listed in Table S15 (Supporting Information). Ten milliliters of the culture were used for RNA isolation.

For cultivation of sigma factor‐overexpressing strains, the mycelium was first inoculated in 50 mL of R5− liquid media in a 250‐mL baffled flask with 8 g of glass beads (3 ± 0.3 diameter) at 30 °C and 200 rpm. R5− liquid media consists of 103 g of sucrose, 10 g of glucose, 5 g of yeast extract, 10.12 g of MgCl_2_·6H_2_O, 0.25 g of K_2_SO_4_, 0.1 g of casamino acids, 5.73 g of TES, 0.28 g of NaOH, 0.08 mg of ZnCl_2_, 0.4 mg of FeCl_3_⋅6H_2_O, 0.02 mg of CuCl_2_⋅2H_2_O, 0.02 mg of MnCl_2_⋅4H_2_O, 0.02 mg of Na_2_B_4_O_7_⋅10H_2_O, and 0.02 mg of (NH_4_)_6_Mo_7_O_24_⋅4H_2_O in 1 L of distilled water. After inoculation, the optical density (OD) at 600 nm was measured, and an appropriate amount of culture (volume [mL] × OD = 0.15) was transferred onto a cellophane membrane (diameter, 8 cm) on 30 mL of R5− solid media (+ 22 g of agar per 1 L of R5− liquid media) in a petri dish with a diameter of 9 cm. Solid culture was performed in triplicate. After 3 days of incubation at 30 °C, the whole cell mass on the cellophane membrane was collected for RNA isolation.

### Construction of Sigma Factor‐Overexpressing Strains

Sigma factors were expressed from a plasmid (pIBR25) with an N‐terminally fused MBP to ensure soluble expression of sigma factors (Figure S1, Supporting Information).^[^ ^40^ ^]^ In some cases, *S. coelicolor* seemed to be sensitive to increased expression of sigma factors, resulting in growth failure. In these cases, the expression strength was tuned using the pSET152 plasmid,^[^ ^41^ ^]^ which is integrated into the genome and thus maintained at a lower copy number than pIBR25. Transformants were not obtained for three sigma factors: SCO4908 (SigQ; related to secondary metabolism and morphological development),^[^ ^42^ ^]^ SCO5243 (SigH; related to morphological development and osmotic stress response),^[^ ^43^ ^]^ and SCO7099 (unknown function) (Table S2, Supporting Information). The sequences of the primers and DNA templates used for plasmid construction are listed in Table S16 (Supporting Information). All PCR procedures were performed using Phusion High‐Fidelity DNA Polymerase (Thermo Scientific, Waltham, MA, USA). To introduce the MBP‐sigma factor expression cassette into the pIBR25 plasmid, the T0 terminator sequence was amplified from the pCRISPomyces‐2 plasmid using primer pairs T0_F_common and T0_R_MluI.^[^ ^44^ ^]^ The PCR product was cloned into the *Xba*I – *Mlu*I site of the pIBR25 plasmid. Next, a codon‐optimized MBP – poly N‐linker construct was synthesized (Integrated DNA Technologies, Coralville, IA, USA) and cloned into the *Bam*HI – *Xba*I site. To introduce the MBP‐sigma factor expression cassette into the pSET152 plasmid, the T0 terminator sequence was amplified from the pCRISPomyces‐2 plasmid using primer pairs T0_F_common and T0_R_PvuI.^[^ ^44^ ^]^ The PCR product was cloned into the *Xba*I – *Pvu*I site of the pSET152 plasmid. The expression cassette cloned into the pIBR25 plasmid was PCR‐amplified using the primer pair ErmE_F and T0_R_MluI and cloned into the *Not*I – *Xba*I site of the T0 terminator cloned into the pSET152 plasmid. Each sigma factor was PCR‐amplified from the genome of *S. coelicolor* using the appropriate primers listed in Table S16 (Supporting Information), and cloned into the *Bcu*I – *Pac*I site of the expression cassette. For the control plasmid expressing only MBP, two primers, Stop_F and Stop_R, were annealed and cloned into the *Bcu*I – *Xba*I site of the expression cassette. The pIBR25‐ and pSET152‐based plasmids were transformed into *S. coelicolor* via protoplast transformation and conjugation, respectively, as previously described.^[^ ^45^ ^]^

### RNA‐seq Library Construction

The cells were resuspended with 1 mL of Sol 1 (25 mm Tris‐HCl pH 8.0, 10 mm EDTA, 50 mm glucose, and 2 mg mL^−1^ lysozyme) and incubated at 30 °C for 10 min (for cells cultivated in cellobiose, the 10‐min incubation was not performed since the cells would have undergone complete lysis if incubated). After incubation, the cells were centrifuged and the supernatant was removed. The cell pellet was resuspended with 500 µL of AE (50 mm sodium acetate [pH 5.2], 10 mm ethylenediaminetetraacetic acid [EDTA]), and 50 µL of 10% sodium dodecyl sulfate solution was added. The cell suspension was then mixed with an equal volume of phenol/chloroform (5:1) solution. The mixture was incubated at 65 °C for 5 min, and RNA was isolated by isopropanol precipitation. Ten micrograms of total RNA were treated with DNase I (New England Biolabs, Ipswich, MA, USA) and cleaned using an RNA Clean & Concentrator Kit (Zymo Research, Irvine, CA, USA) according to the manufacturer's instructions. rRNA was depleted by using NEBNext rRNA Depletion Kit (New England Biolabs) according to manufacturer's instructions or the RiboRid method as previously described, using 1 µg of total RNA.^[^ ^46^ ^]^ For the SigB‐overexpressing strain, RiboRid was performed with the addition of transfer‐messenger RNA‐specific oligo probes (5 pmol/each; Table S17, Supporting Information). RNA‐seq libraries were constructed using a TruSeq Stranded mRNA Library Prep Kit (Illumina, San Diego, CA, USA) according to the manufacturer's instructions. Libraries were sequenced using either Illumina NovaSeq 6000 or HiSeq X Ten systems.

### Data Processing of the RNA‐seq Samples

Raw reads were processed using the CLC Genomics Workbench (CLC Bio, Aarhus, Denmark). Adaptor sequences and low‐quality reads (score < 0.05, number of ambiguous nucleotides > 2, and/or length < 15 nt) were removed. The remaining reads were mapped to the reference genome (accession number: NC003888.3) with the following parameters: mismatch cost = 2, insertion cost = 2, deletion cost = 3, and length fraction = 0.9, and only uniquely mapped reads were saved. After mapping, gene expression was calculated as transcripts per million and log_2_‐transformed. Only genes with open reading frames longer than 100 nt and a maximum raw read count across the RNA‐seq samples > 10 were used for gene expression calculation, and only RNA‐seq samples with a high correlation (Pearson R > 0.95) between biological replicates were retained for conducting ICA. ICA was performed as described previously, and the membership of each iModulon was determined on the basis of the K‐means clustering algorithm.^[^ ^47^ ^]^ The codes are available at https://github.com/SBRG/iModulonMiner. Briefly, RNA‐seq samples generated in‐house in glucose‐defined media were selected as references. The log_2_‐transformed expression of a gene in an RNA‐seq sample was centered toward the glucose‐defined medium condition by subtracting the mean expression of the gene in biological triplicates of the reference condition (Table S4, Supporting Information). ICA was performed by executing the FastICA algorithm,^[^ ^48^ ^]^ implemented in scikit‐learn,^[^ ^49^ ^]^ 100 times with random seeds and a convergence tolerance of 10^−7^. Robust independent components (ICs) were identified by clustering the ICs from the iterated ICA with the DBSCAN algorithm using an epsilon of 0.1 and a minimum cluster seed size of 50.^[^ ^50^ ^]^ The distance matrix was computed using Equation (1) to account for identical components with opposite signs.

(1) $$d_{x,y}=1-∥\rho_{x,y}∥$$

here, ρ_*x*,*y*_ is the Pearson correlation between components *x* and *y*. The final robust ICs were defined as the centroids of the cluster.

Because the number of dimensions selected in ICA can alter the results, the above procedure was applied to each expression profile multiple times with different ranges of the number of dimensions, starting from 20, with a step size of 20. Optimum dimensionality was determined as described previously.^[^ ^47b^ ^]^ Briefly, the number of ICs was monitored with single genes and the number of ICs that were conserved (Pearson R > 0.7) across multiple dimensions (called “final components” or “conserved components”). Two hundred forty was selected as the optimal number of dimensions, where the number of non‐single‐gene ICs was equal to the number of final components in that dimension. Each IC contained a gene weight for each gene (Table S5, Supporting Information), and only genes with weights above a specific threshold were considered members of an iModulon. To determine the threshold, the scikit‐learn implementation of K‐means clustering was applied to the absolute values of the gene weights in each IC using three clusters,^[^ ^49^ ^]^ and all genes in the top two clusters were regarded as significant genes in the iModulon.^[^ ^47a^ ^]^ Transcriptomic dataset, ICA‐decomposed signals, iModulon membership information, and their functional annotations are described in Tables S4–S8 (Supporting Information). In addition, dataset is publicly available through web‐based analysis and visualization tool, iModulonDB (https://imodulondb.org/).^[^ ^51^ ^]^ For annotating smBGC iModulons, smBGCs were predicted by using AntiSMASH (v7.1.0) from the *S. coelicolor* genome (accession number: NC003888.3) with relaxed detection strictness and extra features, including KnownClusterBlast, SubClusterBlast, MIBiG cluster comparison, ActiveSiteFInder, and RREFInder.^[^ ^52^ ^]^

### Motif Analysis

For promoter motif analysis in regulatory iModulons, 50‐nt upstream sequences from transcription start sites were used,^[^ ^8^ ^]^ and if required, 150‐nt upstream sequences from the start codon of operon leaders were also used. The MEME tool, version 5.3.3, in the MEME suite, was used with the following arguments: ‐dna ‐mod zoops ‐minw 15 ‐maxw 45 ‐allw.^[^ ^22^ ^]^

### Actinorhodin Measurement

Solid culture of sigma factor‐overexpressing strains was performed as described above for 3 days, and actinorhodin production was measured as described previously.^[^ ^26^ ^]^ Next, 3 cm × 1 cm × 0.5 cm blocks of the solid culture, including cells and the cellophane membrane, were incubated in 3 mL of methanol at 25 °C for overnight. After incubation, 750 µL of the supernatant was mixed with 250 µL of 4 m KOH and centrifuged at 16 000 × *g* for 1 min. The supernatant was collected, and the concentration of actinorhodin was measured by measuring the absorbance at 640 nm using Tecan Infinite F200 Pro (Tecan Group Ltd., Männedorf, Switzerland).

## Conflict of Interest

The authors declare no conflict of interest.

## Author Contributions

Y.L. and D.C. contributed equally as co‐first authors. B.O.P. and B.‐K.C. conceived and supervised the study. Y.L. and D.C. performed the experiments. Y.L., D.C., B.O.P., and B.‐K.C. analyzed the data and wrote the manuscript. All the authors have read and approved the manuscript.

## Supporting information

Supporting Information

Supplemental Table 1

Supplemental Table 3

Supplemental Table 4

Supplemental Table 5

Supplemental Table 6

Supplemental Table 7

Supplemental Table 8

Supplemental Table 9

Supplemental Table 10

Supplemental Table 12

Supplemental Table 13

## Data Availability

The sequencing data generated in this study are available in the European Nucleotide Archive under the accession numbers PRJEB72243, PRJEB72249, and PRJEB72250.
