# Supplementary material for: Machine‐Learning Analysis of Streptomyces coelicolor Transcriptomes Reveals a Transcription Regulatory Network Encompassing Biosynthetic Gene Clusters
Source: Adv Sci (Weinh). 2024 Sep 12;11(41):2403912. doi: 10.1002/advs.202403912 (PMC11538686; doi:10.1002/advs.202403912)
Supplement: Supplementary file 1 — Supporting Information [file ADVS-11-2403912-s007.pdf]

ADVANCED SCIENCE
Open Access

Supporting Information

for *Adv. Sci.*, DOI 10.1002/advs.202403912

Machine-Learning Analysis of *Streptomyces coelicolor* Transcriptomes Reveals a Transcription Regulatory Network Encompassing Biosynthetic Gene Clusters

Yongjae Lee, Donghui Choe, Bernhard O. Palsson and Byung-Kwan Cho**

Supporting Information

Machine-Learning Analysis of *Streptomyces coelicolor* Transcriptomes Reveals a Transcription Regulatory Network Encompassing Biosynthetic Gene Clusters

Yongjae Lee, Donghui Choe, Bernhard O. Palsson, Byung-Kwan Cho**

Table of Contents

Supplementary Tables

Table S2

Table S11

Table S14

Table S15

Table S16

Table S17

Supplementary Figures

Figure S1

Figure S2

Figure S3

Figure S4

Figure S5

Figure S6

Figure S7

Figure S8

Figure S9

Figure S10

Figure S11

Figure S12

Figure S13

Supplementary References

Table S2 | Sigma factors overexpressed in this study

Locus tag	Gene name	Plasmid	Note
SCO0037	SCO0037	pIBR25	-
SCO0159	SCO0159	pIBR25	-
SCO0194	litS	pIBR25	-
SCO0255	SCO0255	pIBR25	-
SCO0414	SCO0414	pIBR25	-
SCO0632	SCO0632	pIBR25	-
SCO0803	SCO0803	pIBR25	-
SCO0864	SCO0864	pIBR25	-
SCO0866	SCO0866	pIBR25	-
SCO0895	hrdC	pIBR25	-
SCO0942	SCO0942	pIBR25	-
SCO1263	SCO1263	pIBR25	-
SCO1276	sigJ	pIBR25	-
SCO1564	SCO1564	pIBR25	-
SCO1723	SCO1723	pIBR25	-
SCO1876	SCO1876	pIBR25	-
SCO2465	hrdA	pIBR25	-
SCO2639	SCO2639	pIBR25	-
SCO2742	SCO2742	pIBR25	-
SCO2954	sigU	pIBR25	-
SCO3068	sigI	pSET152	-
SCO3202	hrdD	pIBR25	-
SCO3323	bldN	pSET152	-
SCO3356	sigE	pSET152	-
SCO3450	SCO3450	pSET152	-
SCO3613	SCO3613	pSET152	-
SCO3626	SCO3626	pIBR25	-
SCO3715	SCO3715	pIBR25	-
SCO3736	SCO3736	pSET152	-
SCO3892	sigT	pIBR25	-
SCO4005	SCO4005	pIBR25	-
SCO4035	sigF	pSET152	-
SCO4146	SCO4146	pIBR25	-
SCO4409	SCO4409	pIBR25	-
SCO4452	SCO4452	pIBR25	-
SCO4769	sigD	pIBR25	-
SCO4864	SCO4864	pSET152	-

Locus tag	Gene name	Plasmid	Note
SCO4866	SCO4866	pIBR25	-
SCO4895	SCO4895	pIBR25	-
SCO4938	SCO4938	pIBR25	-
SCO4960	SCO4960	pIBR25	-
SCO4996	SCO4996	pSET152	-
SCO5147	SCO5147	pSET152	-
SCO5216	sigR	pIBR25	-
SCO5621	whiG	pIBR25	-
SCO5820	hrdB	pIBR25	-
SCO5934	SCO5934	pSET152	-
SCO6239	SCO6239	pIBR25	-
SCO6520	sigK	pSET152	-
SCO6996	SCO6996	pIBR25	-
SCO7104	SCO7104	pIBR25	-
SCO7105	SCO7105	pIBR25	-
SCO7112	SCO7112	pIBR25	-
SCO7144	SCO7144	pIBR25	-
SCO7192	SCO7192	pIBR25	-
SCO7278	sigL	pIBR25	-
SCO7314	sigM	pIBR25	-
SCO7341	sigG	pSET152	-
SCO0038	SCO0038	pIBR25	SCO0038 is a partial CDS located upstream of SCO0037. Sigma2 domain exists partially in the two CDS, suggesting that functional sigma factor may be translated starting from SCO0038 with a frameshift into the downstream SCO0037. SCO0038-SCO0037 locus was cloned.
SCO0600	sigB	pIBR25	Genomic deletion at both chromosome arms (~SCO1004, SCO7051~)
SCO3709	SCO3709	pIBR25	Genomic deletion at the left chromosome arm (~SCO0384)
SCO4034	sigN	pSET152	Genomic deletion at the left chromosome arm (~SCO0500)
SCO4908	sigQ	-	Failed to express
SCO5243	sigH	-	Failed to express
SCO7099	SCO7099	-	Failed to express

Table S11 | Experimental evidences for the iModulon refined smBGC regions

smBGC	iModulon inferred left boundary			
	Gene	Experiment / Evidence	Note	References
Coelichelin	SCO0489	Gene disruption	No production of coelichelin.<br>SCO3218 is the functional homolog, and only the double disruption mutant abolished the production.	[1]
Flaviolin	SCO1206	Heterologous expression in *E. coli in vitro* characterization	Production of the metabolites confirmed.	[2]
Hydroxyectoine	SCO1864	Gene disruption	Production of ectoine in very low level.	[3]
Desferrioxamine	SCO2780	Gene disruption / complementation	Growth affected in iron limited conditions.	[4]
CDA	SCO3210	-	Function predicted.	[5]
Actinorhodin	SCO5071	Gene disruption	Resulted in reduced production of actinorhodin.	[6]
Albaflavenone	SCO5222	Protein purification & biochemical assay	Function characterized.	[7]
Undecylprodigiosin	SCO5877	Gene disruption	Resulted in reduced production of undecylprodigiosin.	[8]
Geosmin	SCO6073	Gene disruption	No geosmin production	[9]
Coelimycin P1	SCO6268	-	MIBiG addresses coelimycin BGC starting from SCO6265, however, SCO6264-SCO6267 is related to the synthesis of SCB, and SCB is related to the regulation of coelimycin BGC.<br>Experimental evidences for SCO6268's involvement in coelimycin production is lacking.	[10]
SCB	SCO6266	-	MIBiG addresses SCB BGC starting from SCO6265, however, SCO6264 is also involved in the production of SCB.	[10]
SapB	SCO6681	Gene disruption	Results in SapB deficient phenotype.	[11]

smBGC	iModulon inferred right boundary			
	Gene	Experiment / Evidence	Note	References
Coelichelin	SCO0499	Gene disruption	No production of coelichelin.	[12]
Flaviolin	SCO1208	Characterization of the homologous gene of other species	Indirect evidence.	[13]
Hydroxyectoine	SCO1867	Gene disruption	No production of hydroxyectoine.	[3]
Desferrioxamine	SCO2785	Gene disruption	No production of desferrioxamine.	[14]
CDA	SCO3249	Protein purification & biochemical assay	Function characterized.	[15]
Actinorhodin	SCO5092	Gene disruption	No production of actinorhodin.	[16]
Albaflavenone	SCO5223	Protein purification & biochemical assay	Function characterized.	[17]
Undecylprodigiosin	SCO5899	Biochemical assay in crude extract of SCO5899 expressing cells	O-methyltransferase activity confirmed.	[18]
Geosmin	SCO6073	Gene disruption	No geosmin production	[9]
Coelimycin P1	SCO6288	Gene disruption	No coelimycin production	[19]
SCB	SCO6267	Gene disruption	No SCB activity	[10]
SapB	SCO6683	-	SCO6684 also involves in SapB activity.	[20]

Table S14 | Composition of defined media

Defined media stock solutions			
Solution	Component	Amount / 200 mL DW	Note
10X NH_4Cl solution	NH_4Cl	4.84 g	pH was adjusted to 7.0 with KOH and HCl.<br><br>Each solution was autoclaved separately.
10X Na_2SO_4 solution	Na_2SO_4	7.86 g	
10X $MgCl_2$ solution	$MgCl_2$•$6H_2O$	2.03 g	
10X MOPS solution	MOPS	20.93 g	
10X K_2HPO_4 solution	K_2HPO_4	1.00 g	
Carbon source stock solutions			
Solution	Component	Amount / 25 mL DW	Note
10X Arabinose	L-Arabinose	2.50 g	pH was adjusted to 7.0 with KOH and HCl.<br><br>Each solution was filter-sterilized.
10X Cellobiose	D-Cellobiose	2.37 g	
10X DL-malic acid	DL-Malic acid	2.79 g	
10X Fructose	D-Fructose	2.50 g	
10X Fumaric acid	Fumaric acid	2.42 g	
10X Galactose	D-Galactose	2.50 g	
10X Glucose	α-D-Glucose	2.50 g	
10X Glycerol	Glycerol	2.56 g	
10X L-arginine	L-Arginine monohydrochloride	2.92 g	
10X L-glutamic acid	L-Glutamic acid potassium salt monohydrate	3384.16 mg	
10X L-malic acid	L-Malic acid	2.79 g	
10X L-Proline	L-Proline	1.92 g	
10X Maltose	Maltose	2.37 g	
10X Mannitol	D-Mannitol	2.53 g	
10X Mannose	D-Mannose	2.50 g	
10X Ribose	D-Ribose	2.50 g	
10X Succinic acid	Succinic acid	2.46 g	
10X Tween 20	Tween 20	1.76 g	
10X Tween 80	Tween 80	1.70 g	
10X Xylose	D-Xylose	2.50 g	
Trace element solution			
Solution	Component	Amount / 1 L DW	Note
Trace element solution	$ZnCl_2$	0.40 g	The solution was autoclaved.
	$FeCl_3$•$6H_2O$	2.00 g	
	$CuCl_2$•$2H_2O$	0.10 g	
	$MnCl_2$•$4H_2O$	0.10 g	
	$Na_2B_4O_7$•$10H_2O$	0.10 g	
	$(NH_4)_6Mo_7O_{24}$•$4H_2O$	0.10 g	
Defined media			
Solution	Component	Amount for 1X concentrate	Note
Defined media	10X NH_4Cl solution	5 mL	For phosphate limitation samples, glucose was used as the carbon source. The amount of distilled water and 10X K_2HPO_4 solution was adjusted for desired phosphate concentration.
	10X Na_2SO_4 solution	5 mL	
	10X $MgCl_2$ solution	5 mL	
	10X MOPS solution	5 mL	
	10X K_2HPO_4 solution	5 mL	
	Carbon stock solution	5 mL	
	Trace element solution	10 µL	
	DW	20 mL	

Table S15 | Sampling condition for the defined media

Condition	Initial O.D. at 600 nm	Culture period	Sampling O.D. at 600 nm (biological triplicate)		
Arabinose	0.01	40 hr	0.440	0.340	0.360
Glucose	0.01	40 hr	0.580	0.580	0.500
Ribose	0.01	40 hr 45 min	4.580	4.240	4.680
Tween 80	0.01	90 hr 15 min	0.314	0.292	0.309
Cellobiose	0.05	23 hr 30 min	2.975	3.065	3.215
DL-malic acid	0.05	23 hr 30 min	0.303	0.295	0.328
Fructose	0.05	40 hr 45 min	0.600	0.632	0.672
Fumaric acid	0.05	21 hr	0.810	0.738	0.766
Galactose	0.05	21 hr 15 min	1.765	1.550	1.880
Glycerol	0.05	19 hr 45 min	1.416	1.454	1.316
L-arginine	0.05	44 hr	1.470	1.395	1.225
L-glutamic acid	0.05	43 hr 15 min	1.252	1.044	1.040
L-malic acid	0.05	46 hr 45 min	3.440	3.610	3.640
L-Proline	0.05	44 hr	0.536	0.514	0.402
Maltose	0.05	19 hr 45 min	0.700	0.702	0.678
Mannitol	0.05	19 hr 45 min	0.504	0.460	0.494
Mannose	0.05	45 hr 45 min	4.250	4.020	3.740
Succinic acid	0.05	47 hr 15 min	1.155	0.930	1.006
Tween 20	0.05	46 hr 45 min	0.400	0.374	0.380
Xylose	0.05	21 hr 15 min	0.535	0.461	0.406
Phosphate 2X	0.01	21 hr	3.565	3.280	4.060
Phosphate 1X	0.01	21 hr	3.670	3.815	3.280
Phosphate 1/5X	0.01	21 hr	2.830	2.910	2.720
Phosphate 1/10X	0.01	21 hr	1.580	1.710	1.725
Phosphate 1/30X	0.01	21 hr	0.845	0.740	0.740

Table S16 | List of primers and DNA templates used in this study

Name	Sequence (From 5′ to 3′)
codon optimized MBP – poly N linker	atcgttgactgcttacagggatccctgcagtctaaaaaggaggcggagatgaagatcgaggagggcaagctggtgatctggatcaacggcgacaagggctacaacggtctcgccgaggtagggaagaaattcgagaaggacaccggcatcaaggtgacggtggagcacc ccgacaagttggaggagaagttcccgcaggtcgccgccaccggagacggccccgacatcatcttctgggcccacgaccggttcggcggctacgcccagtcgggactgctcgccgagatcaccccggacaaggccttccaggacaagctctacccgttcacgtgggacgc ggtccgctacaacggtaagctgatcgcctacccgatcgccgtcgaggccctcagcctgatctacaacaaagatctgctgcccaacccgccgaagacctgggaggagatcccggcgctggacaaggaactgaaggcgaagggcaagtcggccctgatgttcaacctccagg agccgtacttcacctggcccctcatcgcggccgacgggggctatgcattcaagtacgagaacggcaagtacgacatcaaggacgtcggcgtggacaacgccggcgccaaggcaggcctgaccttcctggtcgacctgatcaagaacaagcacatgaacgcggacacgga ctactccatcgccgaagcggccttcaacaagggcgagaccgcgatgaccatcaacgggccctgggcctggtcgaacatcgacaccagcaaggtcaactacggcgtcaccgtgctgccgacgttcaaggggcagccctccaagcccttcgtcggggtcctctccgccggcat caatgccgcttcaccgaacaaggagctggcgaaggagttcctcgaaaactacctgctcaccgacgagggggctggaggcggtgaacaaggacaagccgctgggtgccgtggcgctcaagtcctacgaggaggaactcgccaaggacccgcgcatcgcggcgacgatgga gaacgcgcagaagggcgagatcatgccgaacatcccccagatgtcggcgttctggtacgcggtgcgcacggccgtcatcaacgccgcctccggccggcagaccgtcgacgaggccctgaaggacgcccagaccaactccagcagcaacaacaacaacaataacaataa caacaacctgggtctcgaggactacgacatacccaccacgcccggcgagaacctgtacttccagggcactagtcatatgttaattaaacagcttctagattcgctattgcctccata

Name	Sequence (From 5′ to 3′)	Name	Sequence (From 5′ to 3′)	Name	Sequence (From 5′ to 3′)
T0_F_common	cgcatctagactggatttgttcagaacg	SCO2954_F	cgtgactagtatgggcgtgcgcaaggatt	SCO4908_PacI_R	acgcttaattaatcaggcgaccagttcgc
T0_R_MluI	tccgacgcgtgataaaaaacgcccggcg	SCO2954_R	cgacttaattaatcacgccgtcaccccc	SCO4938_BcuI_F	cgatactagtatgaccacccgatccgcg
T0_R_PvuI	taggcgatcggataaaaaacgcccggcg	SCO3068_F	tgccactagtatgtcaccccggctcgac	SCO4938_PacI_R	acgcttaattaatcacgcggccggggcc
ErmE_F	attagcggccgcgacggccagtgaattaa	SCO3068_R	acgcttaattaatcactcctcgaccgtgag	SCO4960_BcuI_F	cgatactagtatgcgagaacggcatgcg
SCO0037_F	tgctactagtatggcagaaggccgaccg	SCO3202_F	tctgactagtatggcaacccgtgccgtc	SCO4960_PacI_R	acgcttaattaatcatggcgtcgccgcc
SCO0037_R	agccttaattaatcagcagctcgacgacgc	SCO3202_R	agccttaattaatcaggccgccgcctcgaa	SCO4996_BcuI_F	cgatactagtatgaggcaggggcggg
SCO0038_F	tcgcactagtatgcacgcaggccaggac	SCO3323_BcuI_F	cgatactagtatggagctggtcgagcg	SCO4996_PacI_R	acgcttaattaactagggtctggcgtactc
SCO0159_BcuI_F	cgatactagtatgatccgcgtggacagca	SCO3323_PacI_R	acgcttaattaatcagcgggcgtcgtcg	SCO5147_BcuI_F	cgatactagtatgaacgacaccgctgct
SCO0159_PacI_R	acgcttaattaatcaccactgggcgatgag	SCO3356_BcuI_F	cgatactagtatgggcgaggtgctcga	SCO5147_PacI_R	acgcttaattaatcacgcgctcgtgccc
SCO0194_BcuI_F	cgatactagtatgaacacgcgtacacga	SCO3356_PacI_R	acgcttaattaatcaggccgcgcaacgc	SCO5216_F	agtgactagtatgggtccggtcactggga
SCO0194_PacI_R	acgcttaattaatcagacacacgtgtcatgtg	SCO3450_BcuI_F	cgatactagtatggaatgcgggtcaggc	SCO5216_R	agcgttaattaatcatgaccccgagccttt
SCO0255_BcuI_F	cgatactagtatgcccgagggcatcctca	SCO3450_PacI_R	acgcttaattaatcacatcacacctcgctt	SCO5243_BcuI_F	cgatactagtatgagggacggggacggg
SCO0255_PacI_R	acgcttaattaatcagccccgggcgggga	SCO3613_BcuI_F	cgatactagtatgccggtgatcgcgc	SCO5243_PacI_R	acgcttaattaattactcctcgacgagcag
SCO0414_BcuI_F	cgatactagtatggggacggctgggac	SCO3613_PacI_R	acgcttaattaatcatgcgggagcctcca	SCO5621_F	tgcgactagtatgccccagcacacctcc
SCO0414_PacI_R	acgcttaattaatcagcctacccacggcc	SCO3626_BcuI_F	cgatactagtatgcccgcacgatccgac	SCO5621_R	agtcttaattaatcagcggccgaaacccg
SCO0600_F	tcgcactagtatgacgacgacgaccgcg	SCO3626_PacI_R	acgcttaattaatcagcctatgcccgtgat	SCO5820_F	agcgactagtatgtcggccagcacatccc
SCO0600_R	cgtgttaattaatcaggtggtgctgagcat	SCO3709_F	agtgactagtatgcgaccaccagacgaa	SCO5820_R	agcgttaattaatcagtcgaggtagtcgcg
SCO0632_BcuI_F	cgatactagtatgaaggaagccgtgcac	SCO3709_R	agtcttaattaatcagccatgtcgcacctc	SCO5934_BcuI_F	cgatactagtatgtccgaccgggagctg
SCO0632_PacI_R	acgcttaattaatcacgcgctcacccca	SCO3715_BcuI_F	cgatactagtatgctcaccaacctgtgc	SCO5934_PacI_R	acgcttaattaatcatcgtgcgacctcctt
SCO0803_BcuI_F	cgatactagtatgcggggacagggcga	SCO3715_PacI_R	acgcttaattaatcacgggcacggaccac	SCO6239_BcuI_F	cgatactagtatgccggaagacacacct
SCO0803_PacI_R	acgcttaattaatcaggcctccagcccgga	SCO3736_BcuI_F	cgatactagtatgaaacggtcccgtgac	SCO6239_PacI_R	acgcttaattaatcagtcgagtggccaatt
SCO0864_BcuI_F	cgatactagtatgacccctgccctgccc	SCO3736_PacI_R	acgcttaattaatcaaggcgtgtggtcaag	SCO6520_BcuI_F	cgatactagtatgccgatccacgccagc
SCO0864_PacI_R	acgcttaattaatcaggccgcggcccgctc	SCO3892_BcuI_F	cgatactagtatggcgggcggcgcga	SCO6520_PacI_R	acgcttaattaatcacgcctgcggaccc
SCO0866_BcuI_F	cgatactagtatgatcagtccagccctgt	SCO3892_PacI_R	acgcttaattaatcacgctcgtccacctcc	SCO6996_BcuI_F	cgatactagtatggaccgtgcagatgtcg
SCO0866_PacI_R	acgcttaattaatcaggccgaagccctctg	SCO4005_BcuI_F	cgatactagtatgaaggcacgccagccg	SCO6996_PacI_R	acgcttaattaatcatttcgtcccccgtgc
SCO0895_BcuI_F	cgatactagtatggcgcccacggcacg	SCO4005_PacI_R	acgcttaattaattaccgaacgaaccccag	SCO7099_BcuI_F	cgatactagtatgggtggaaccggtgac
SCO0895_PacI_R	acgcttaattaatcagctcgcccagtcca	SCO4034_BcuI_F	cgatactagtatgtccgcagaacagggc	SCO7099_PacI_R	acgcttaattaatcacacgtgcccgacc
SCO0942_BcuI_F	cgatactagtatgacggccggaatcacc	SCO4034_PacI_R	acgcttaattaatcagtcggagatgagacc	SCO7104_BcuI_F	cgatactagtatggagccggtggttga
SCO0942_PacI_R	acgcttaattaatcaccgcaggtcggcgg	SCO4035_BcuI_F	cgatactagtatgccggccagtactgcg	SCO7104_PacI_R	acgcttaattaatcaggcgcgagggcgg
SCO1263_BcuI_F	cgatactagtatgaccaccgacaccgcga	SCO4035_PacI_R	acgcttaattaattatgcgtcgatccggtt	SCO7105_BcuI_F	cgatactagtatggcaggcgacctgagc
SCO1263_PacI_R	acgcttaattaatcagagaacaccggccag	SCO4146_BcuI_F	cgatactagtatgagcgaaacgagaagcg	SCO7105_PacI_R	acgcttaattaatcagacgcagagggccgc
SCO1276_BcuI_F	cgatactagtatggccgaacaaatggaa	SCO4146_PacI_R	acgcttaattaatcatgccagcgcctccc	SCO7112_BcuI_F	cgatactagtatgcacgctggcgccc
SCO1276_PacI_R	acgcttaattaattatcgggcgcagccttc	SCO4409_BcuI_F	cgatactagtatggcgaagagagacgtg	SCO7112_PacI_R	acgcttaattaatcagtcccgcagggaagc
SCO1564_BcuI_F	cgatactagtatgacgcacgacctggtc	SCO4409_PacI_R	acgcttaattaatcatgccgcacccccgta	SCO7144_BcuI_F	cgatactagtatgtccgcgcccagcc
SCO1564_PacI_R	acgcttaattaatcagcccgccgcaacct	SCO4452_BcuI_F	cgatactagtatggacgaggccctggtc	SCO7144_PacI_R	acgcttaattaatcacgcggcctgacggg
SCO1723_F	tctgactagtatggcgggcgaccgggtg	SCO4452_PacI_R	acgcttaattaatcaccggcgcccacgg	SCO7192_BcuI_F	cgatactagtatgggcgccaagagggtt
SCO1723_R	agcgttaattaatcatgcgacgccgcccag	SCO4769_BcuI_F	cgatactagtatgcgtgacgacgatgcg	SCO7192_PacI_R	acgcttaattaatcatgcggcctcgtagtc
SCO1876_BcuI_F	cgatactagtatggggacagtcgtcgac	SCO4769_PacI_R	acgcttaattaactactgctccgccagcg	SCO7278_BcuI_F	cgatactagtatgcagaccgccgtggtc
SCO1876_PacI_R	acgcttaattaatcaccgggcccctccc	SCO4864_BcuI_F	cgatactagtatgaccttggggggcgc	SCO7278_PacI_R	acgcttaattaatcaggcgcagccgagttc
SCO2465_BcuI_F	cgatactagtatgcgaggcggacagcg	SCO4864_PacI_R	acgcttaattaatcatgtcgactcccccag	SCO7314_BcuI_F	cgatactagtatgctcatagaaacgccc
SCO2465_PacI_R	acgcttaattaatcagtccaggtagcccct	SCO4866_BcuI_F	cgatactagtatgctgggggacgacgc	SCO7314_PacI_R	acgcttaattaatcacctgctgtgtcccct
SCO2639_BcuI_F	cgatactagtatgacgtacgcctcgcgc	SCO4866_PacI_R	acgcttaattaatcacatgtccttctgcgt	SCO7341_BcuI_F	cgatactagtatgcgtgtcgccggcaga
SCO2639_PacI_R	acgcttaattaatcacaccacctcgtcctc	SCO4895_BcuI_F	cgatactagtatgaccaacggcaccgtg	SCO7341_PacI_R	acgcttaattaatcagcgggcctccgcc
SCO2742_BcuI_F	cgatactagtatgcacgagacaccgccg	SCO4895_PacI_R	acgcttaattaactactggacctcgtcggc	Stop_F	ctagttgattaattaaacagctt
SCO2742_PacI_R	acgcttaattaatcaccggctcaggctcg	SCO4908_BcuI_F	cgatactagtatgacggtggttgacgcg	Stop_R	ctagaagctgtttaattaatcaa

Table S17 | Oligo probes for tmRNA

Name	Sequence (From 5′ to 3′)	Length
tmRNA_1	ggaatcgaacccgcgtccaacggtgcggaatc	32 mer
tmRNA_2	tccgtgtgcagtccgcttcgattttctcggcc	32 mer
tmRNA_3	cacgcggacaagtctccgacgggcccagtcac	32 mer
tmRNA_4	ttccctcttcaccccgtgaccgggatcaaggt	32 mer
tmRNA_5	ctagctgatgccaggatccgggtcgggaacag	32 mer
tmRNA_6	tgacactcccttagtaggaggctcgcttcgct	32 mer
tmRNA_7	tcaggcagcgagggcgaaggcctgctgggagg	32 mer
tmRNA_8	gcgcttggtgttggcgattatttttcggcct	32 mer
tmRNA_9	ggtttacgagatcatggccgcttcctcgacac	32 mer
tmRNA_10	ttcccctgcttcgacagccgctgtcgaaaccg	32 mer

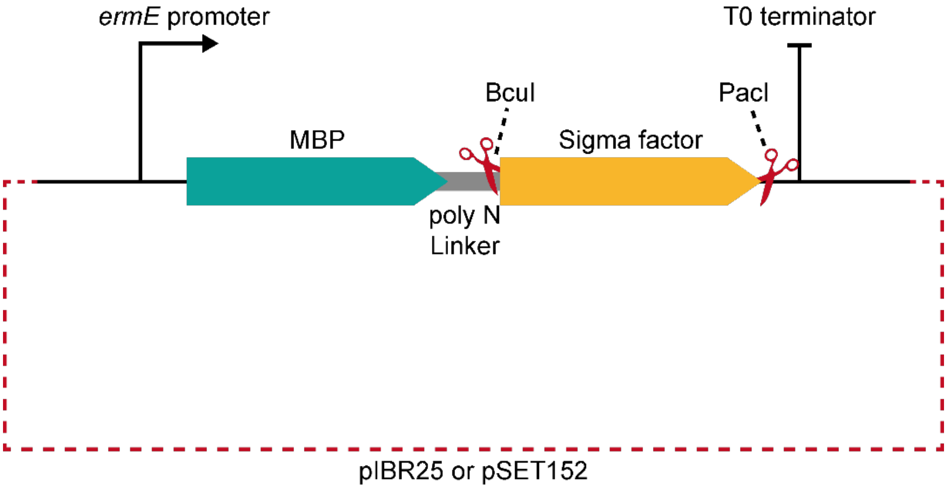

Figure S1. The sigma factor overexpression plasmid used in this study. Each sigma factor was expressed under the *ermE* promoter. Maltose binding protein (MBP) was N-terminally fused for soluble expression. The sequence of the expression cassette is identical regardless of the backbone plasmid.

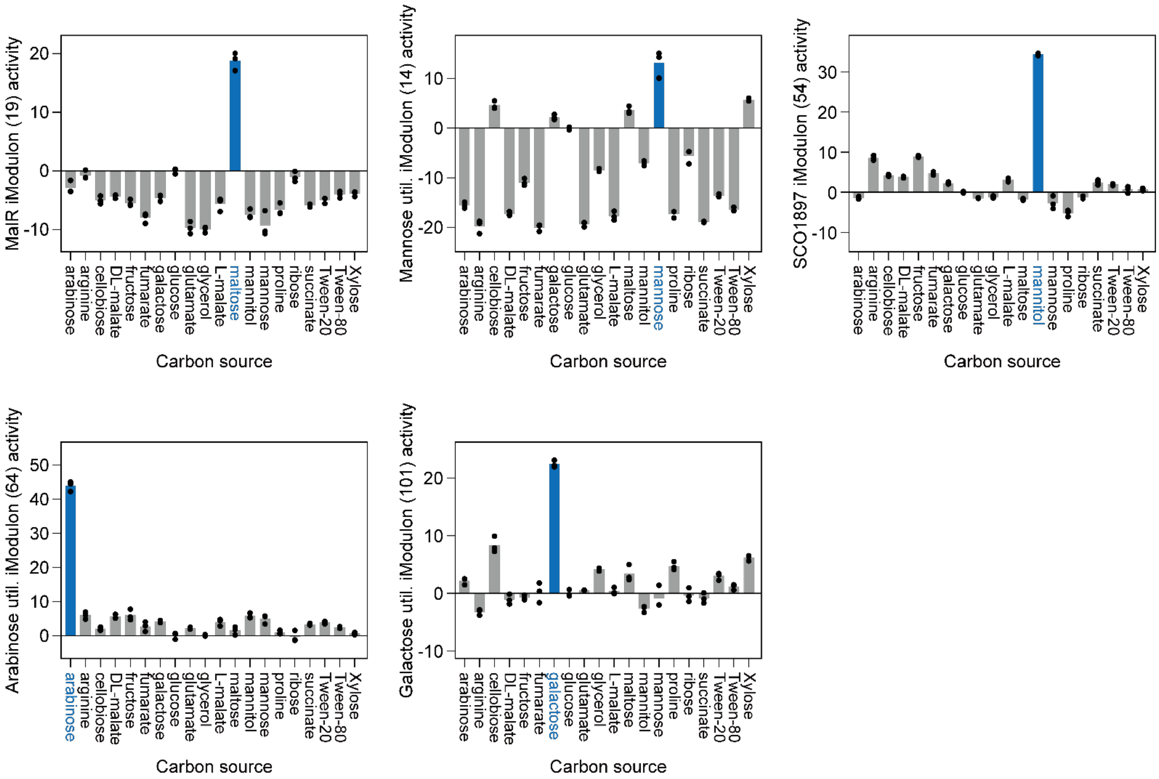

Figure S2. Activities of catabolic iModulons in when cells are given with different sugars as a sole carbon source. Dots are individual transcriptomic samples.

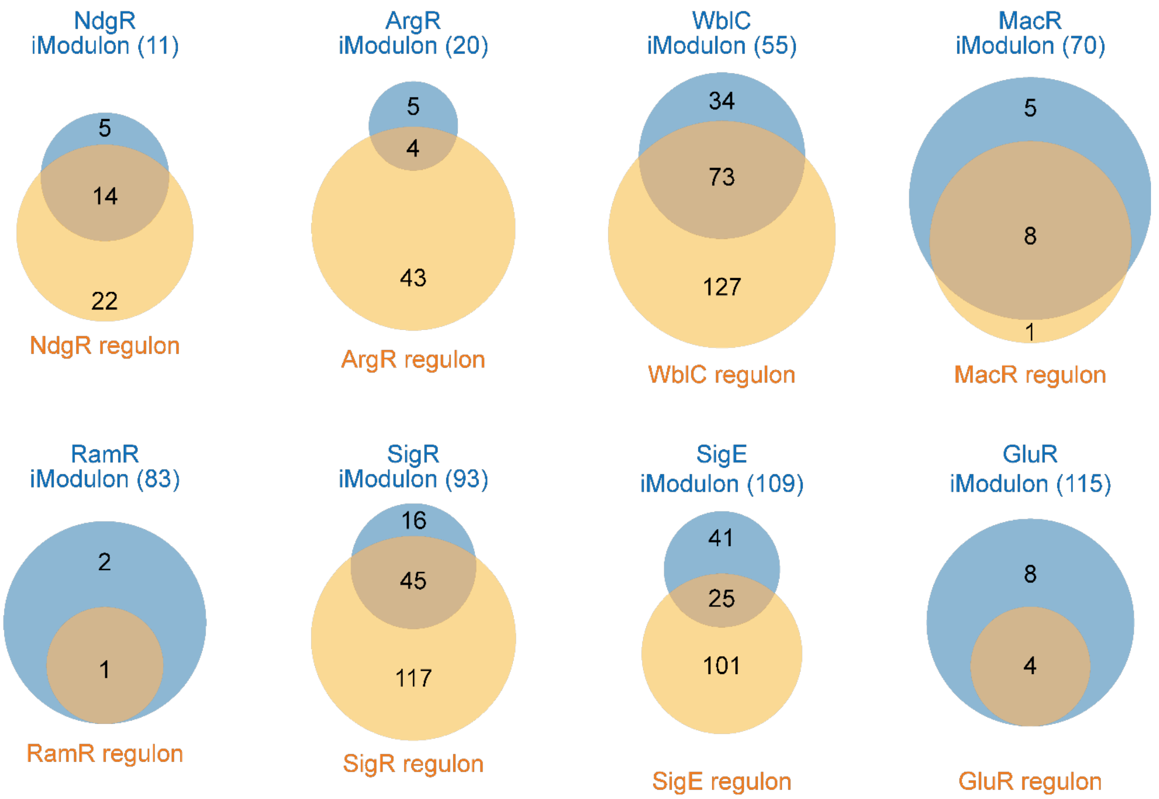

Figure S3. Comparison of iModulon members and known regulatory information. Only the known regulations with direct regulator binding evidences were considered. Regulon information was pulled from previous reports.[21]

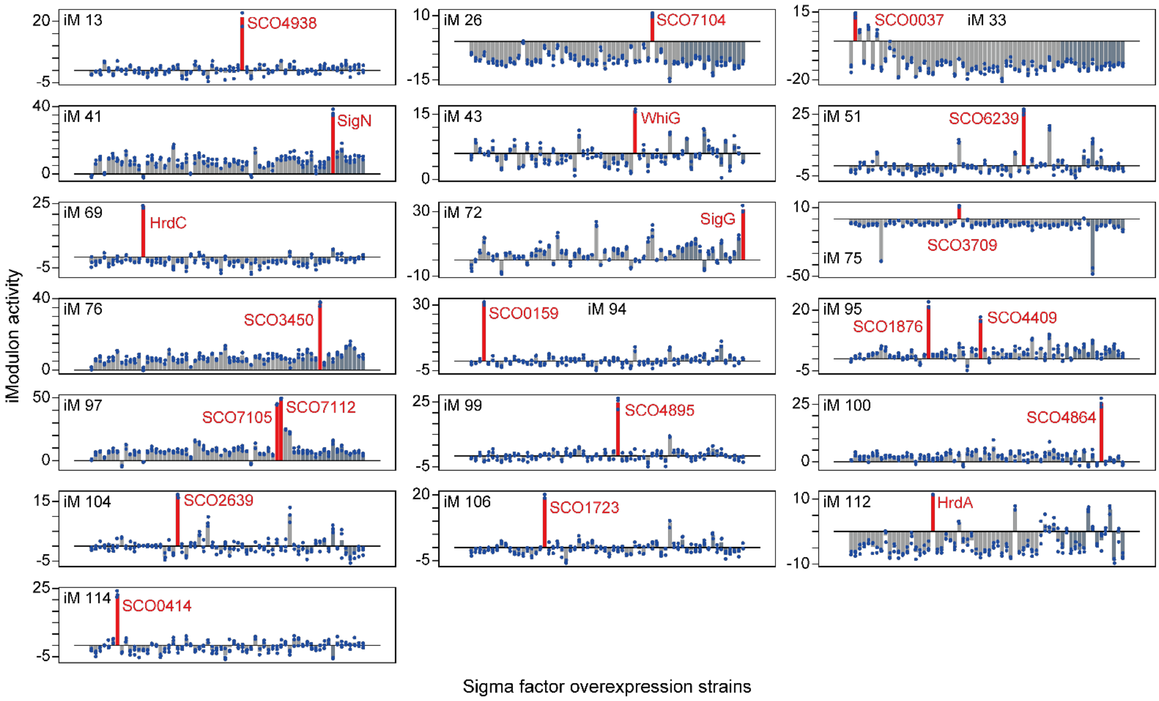

Figure S4. iModulons exhibiting specific activities among sigma factor overexpression strains. Dots are individual transcriptomic samples.

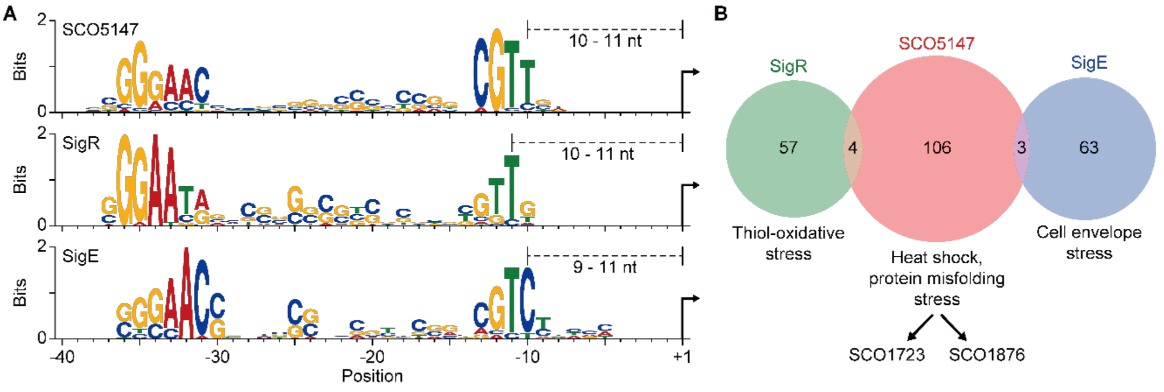

Figure S5. Comparison of iModulons for stress response related sigma factors. A) The promoter motif found in each iModulon using MEME suite.[22] Arrows indicate transcription start sites.[23] B) Venn diagram showing the gene membership of the three iModulons.

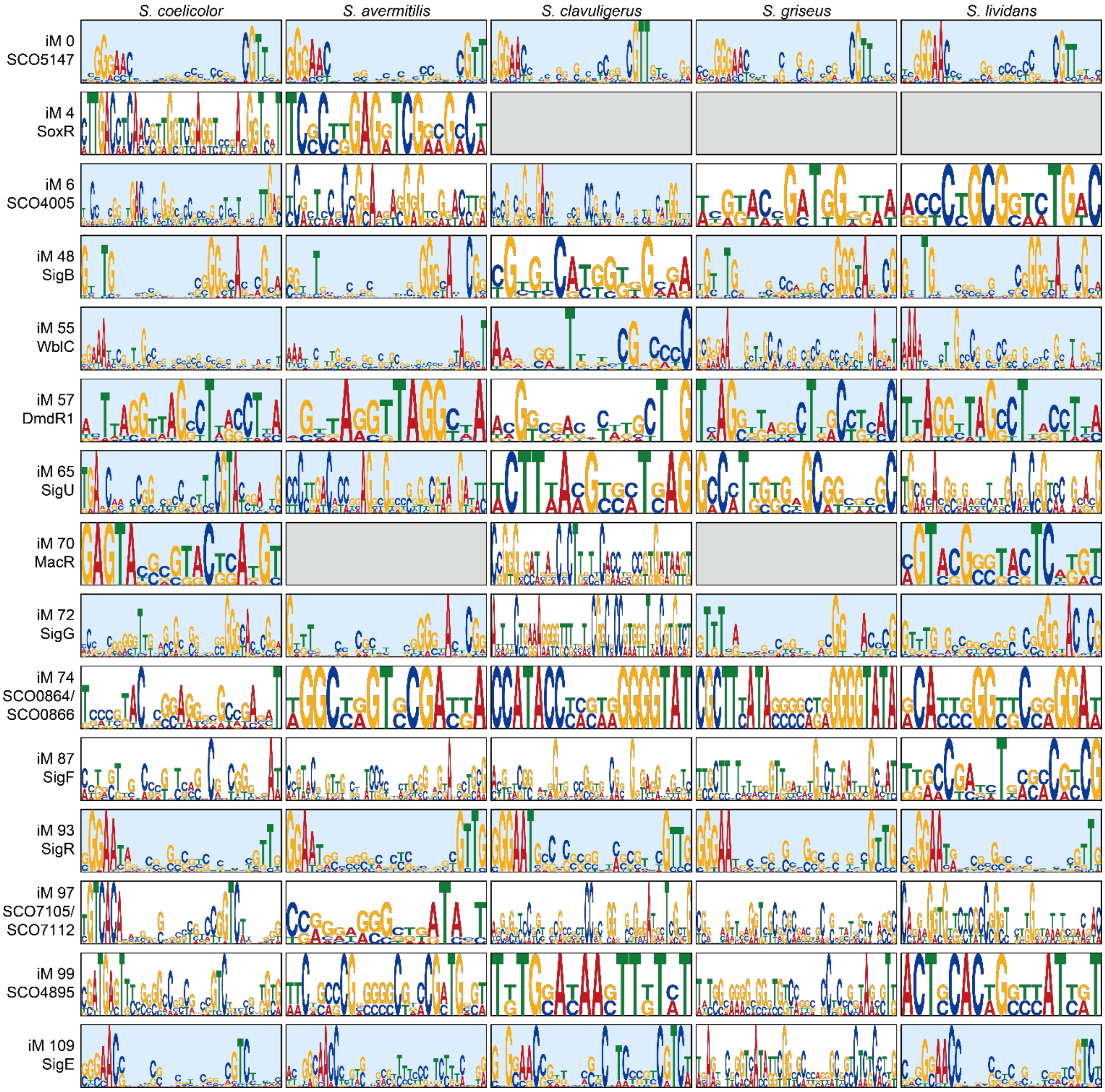

Figure S6. Sequence elements conserved in the homologs of regulatory iModulons. Motifs with blue colored background indicates the presence of sequence homology to the *S. coelicolor*.

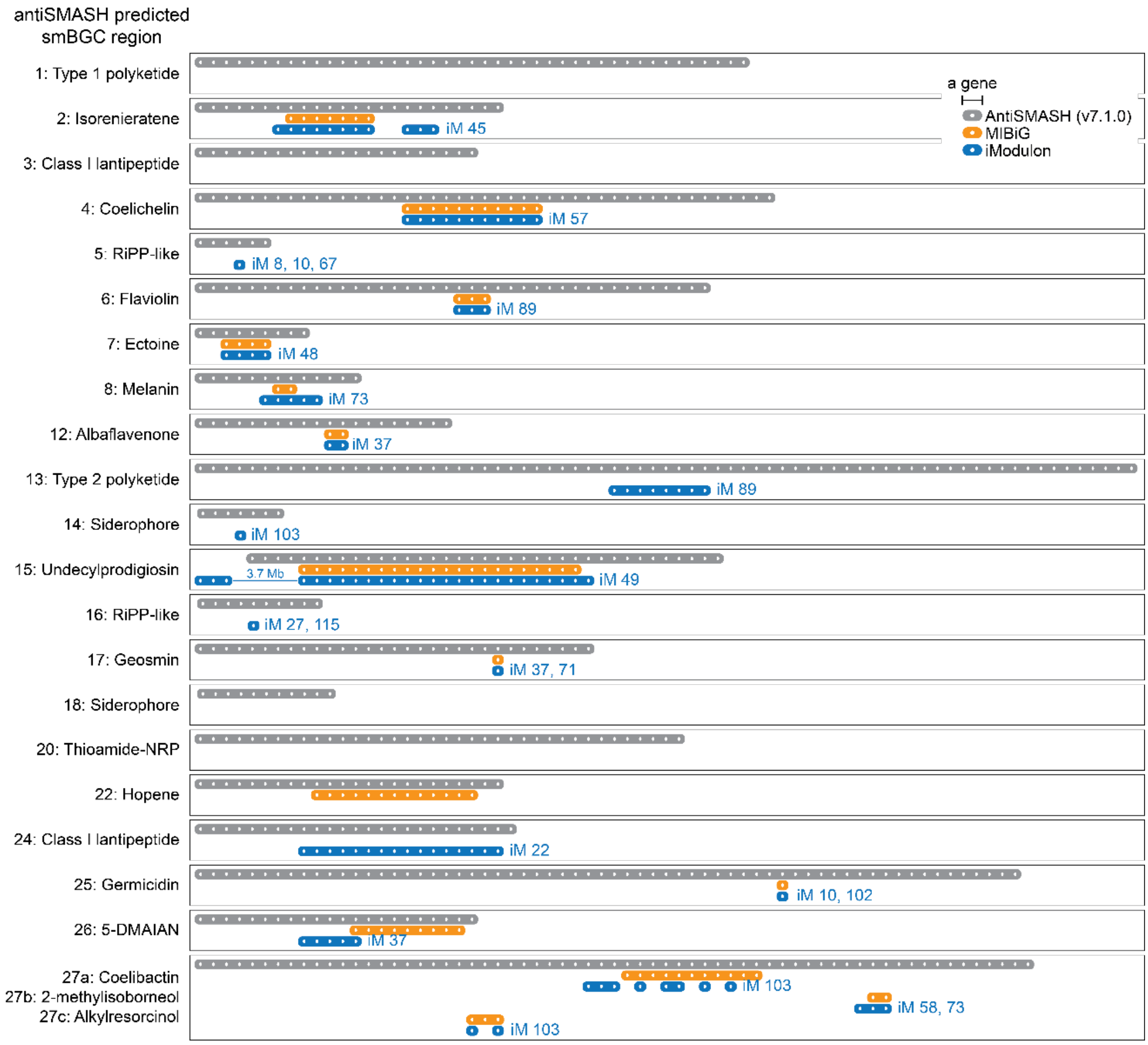

Figure S7. Biosynthetic gene clusters (BGCs) identified by AntiSMASH, MIBiG database, and ICA analysis. Each dot represents a gene. Gray, orange, iModulon strips are BGCs predicted by AntiSMASH, MIBiG, and iModulon, respectively. RiPP: ribosomally synthesized and post-translationally modified peptide. 5-DMAIAN: 5-dimethylallylindole-3-acetonitrile.

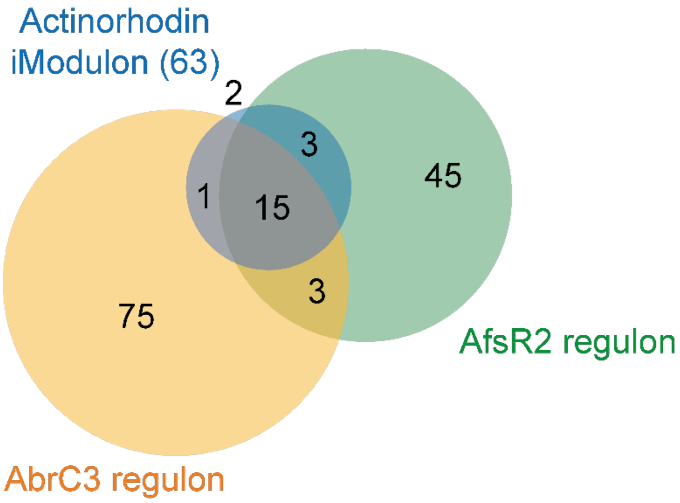

Figure S8. Venn diagram between the genes in actinorhodin iModulon and genes affected by AbrC3 and AfsR2. Regulatory information of AbrC3 and AfsR2 was pulled from previous reports.[21b]

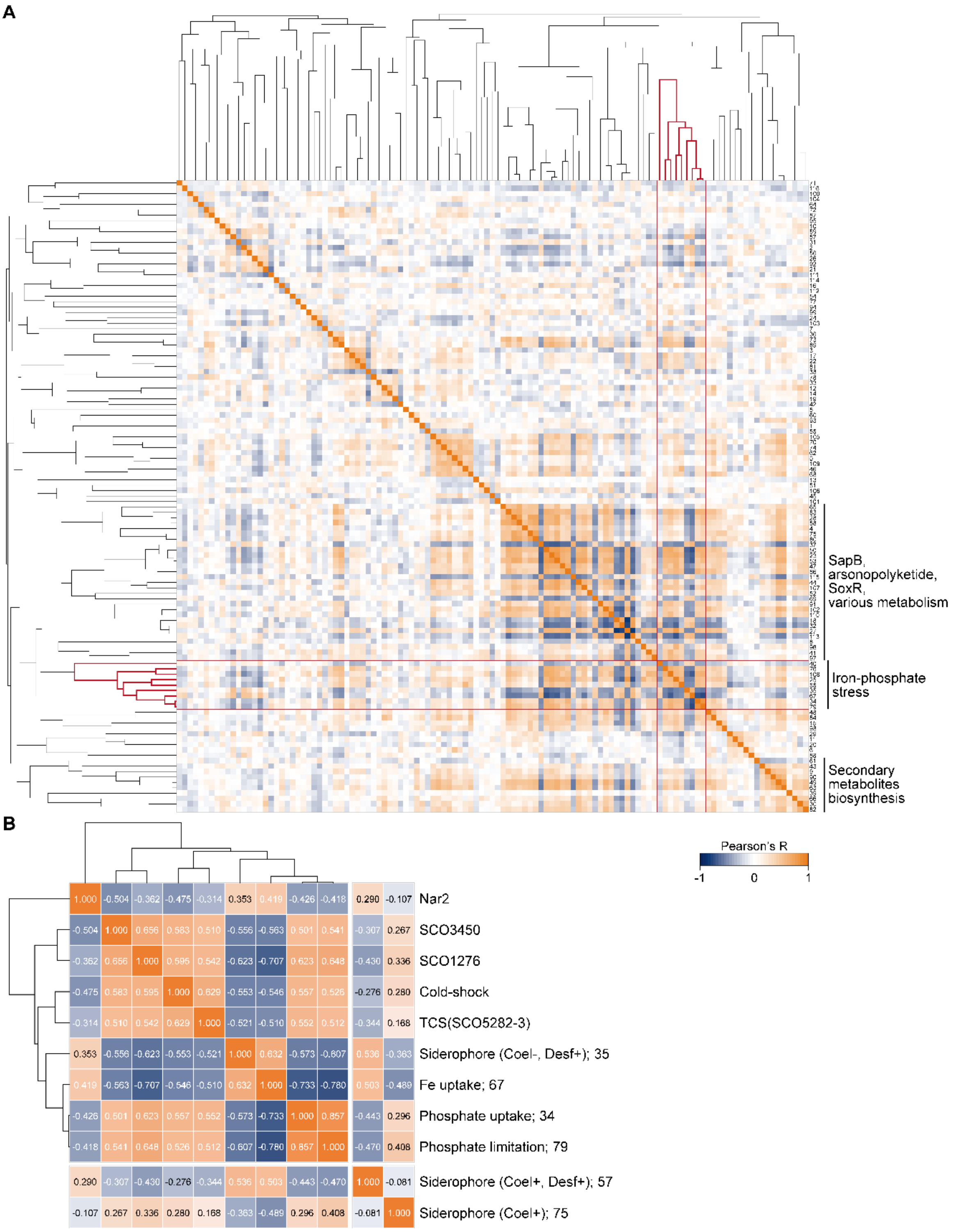

Figure S9. Hierarchical clustering of activity correlations between iModulons across the experimental conditions. A) Hierarchical clustering results of all iModulons. B) Enlarged view of the stress response related cluster next to the biosynthetic iModulons.

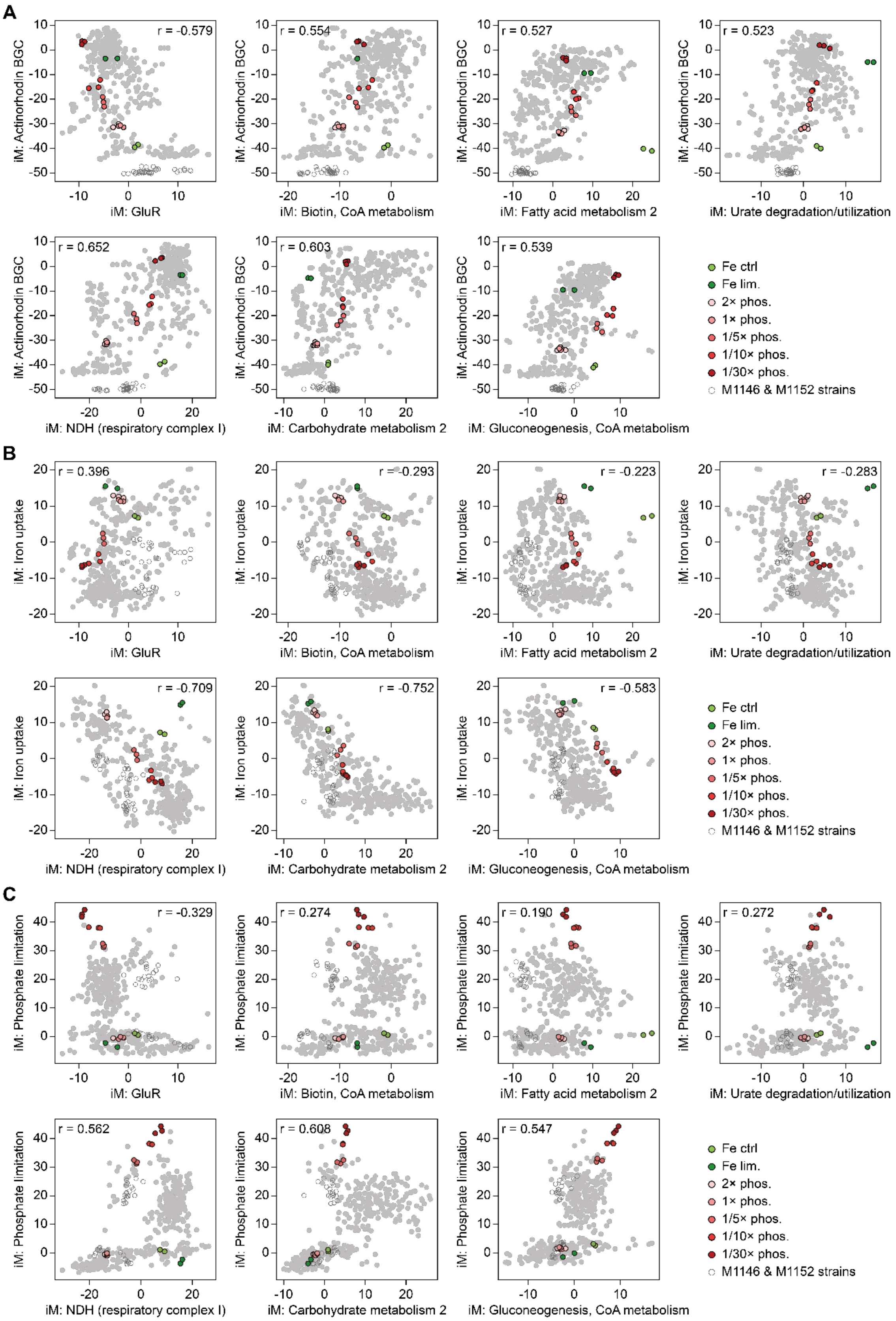

Figure S10. Effect of phosphate and iron stress on metabolic iModulons related to actinorhodin biosynthesis. A) Metabolic iModulons coordinated with actinorhodin iModulon (|Pearson's r| > 0.5). Correlation of the actinorhodin related metabolic iModulons with B) iron uptake iModulon and C) phosphate limitation iModulon.

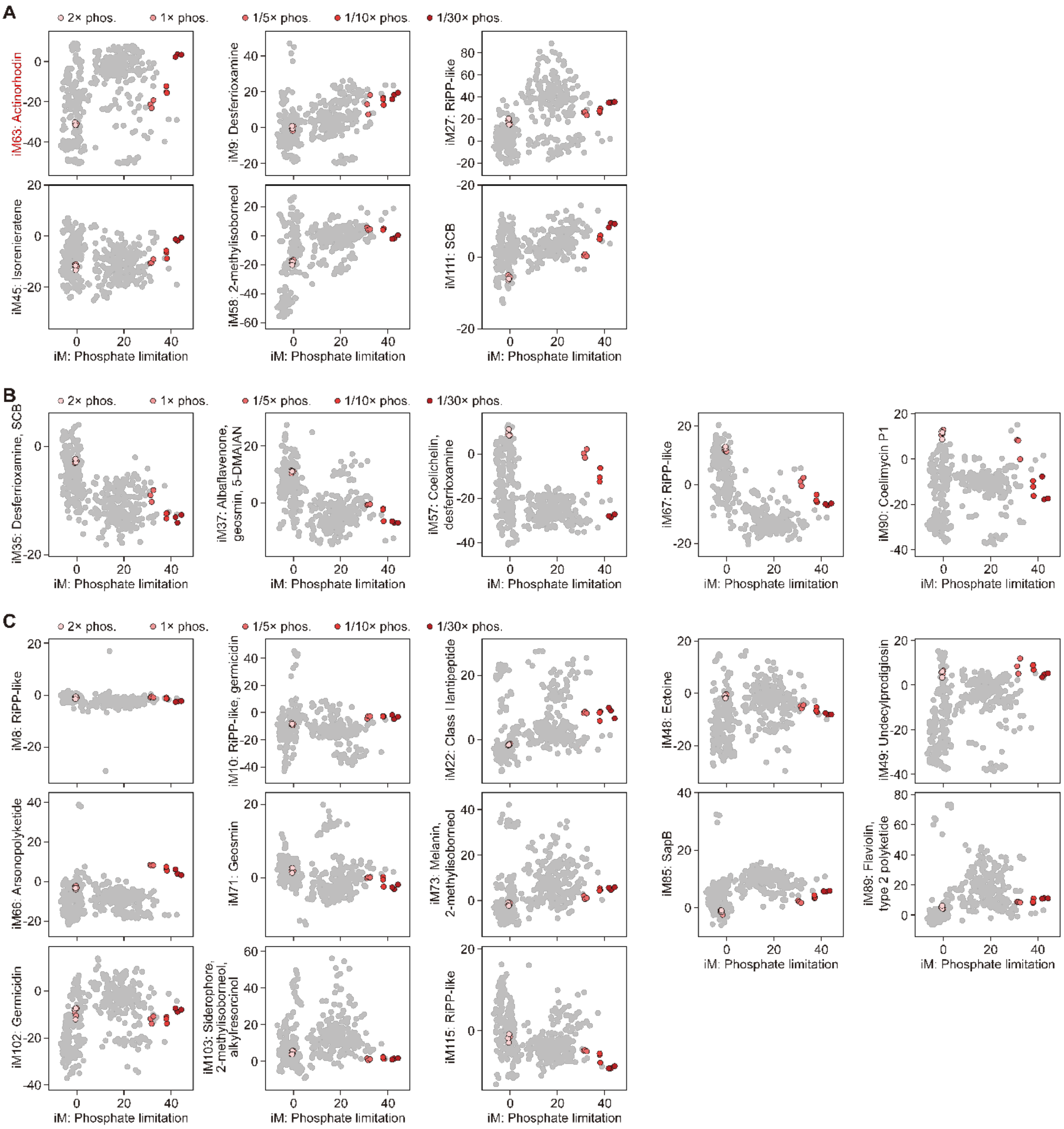

Figure S11. Effect of phosphate stress on secondary metabolite biosynthetic iModulons. Scatter plots show iModulons A) activated or B) repressed by phosphate limitation. C) Activities of many iModulons did not respond to phosphate stress.

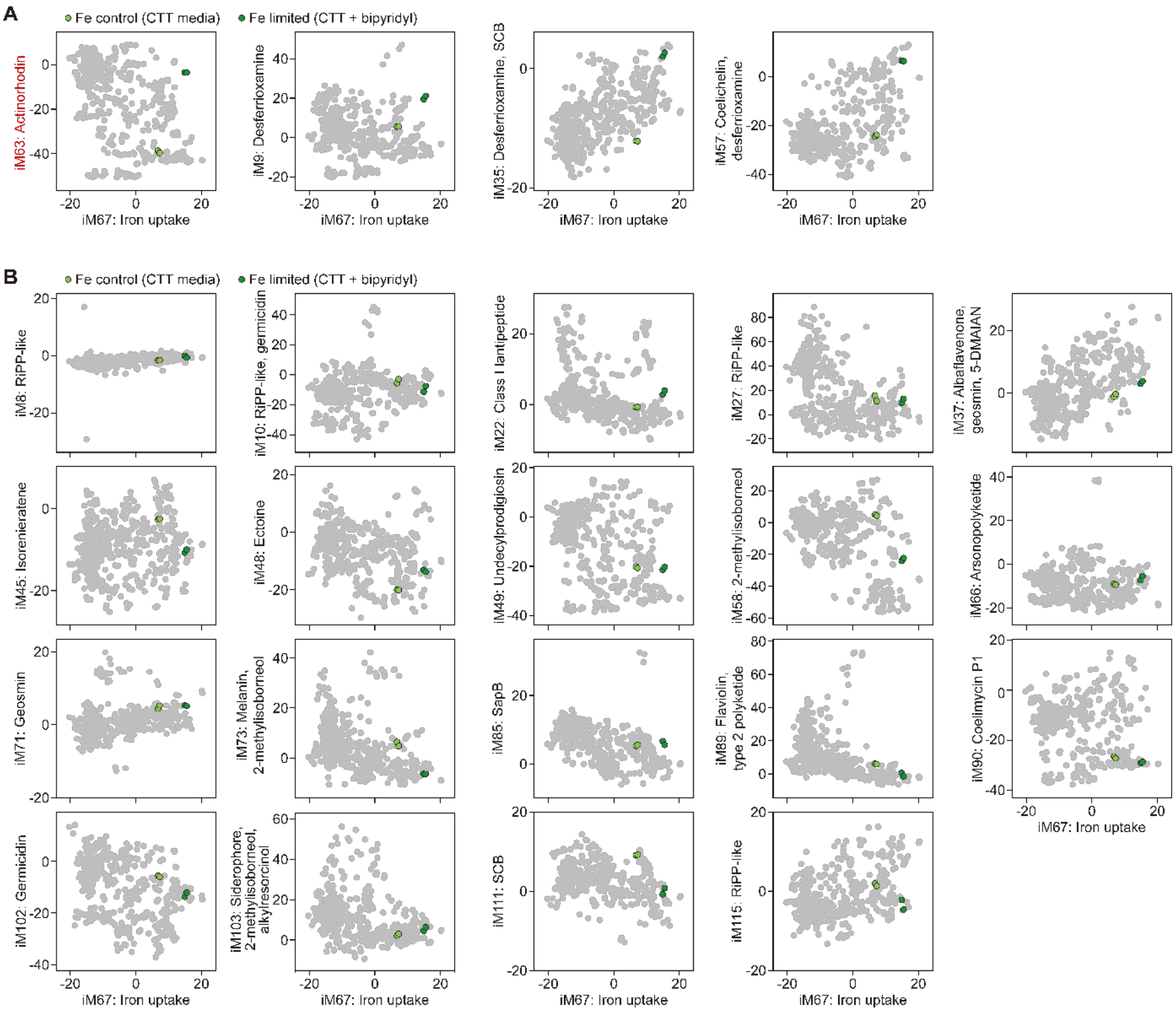

Figure S12. Effect of iron stress on secondary metabolite biosynthetic iModulons. A) Actinorhodin and siderophore biosynthetic iModulons were activated by iron limitation. B) Majority of iModulons were not related to iron stress or had negatively affected by iron limitation.

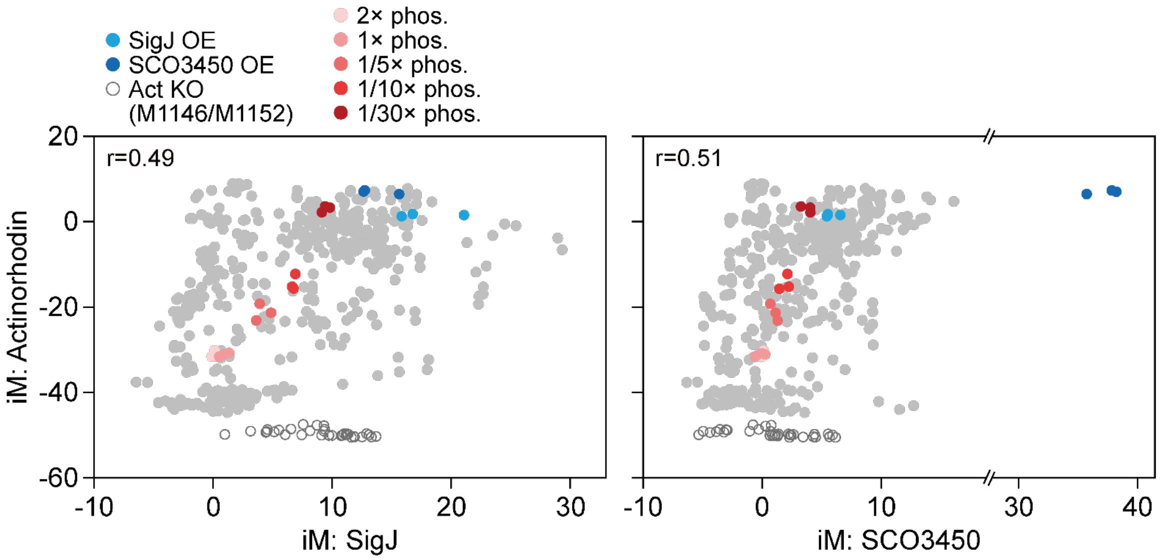

Figure S13. Activity correlation between SigJ and SCO3450 iModulons with actinorhodin iModulon. r is the Pearson's correlation coefficient excluding knockout strains.

Supplementary References

[1] S. Lautru, D. Oves-Costales, J. L. Pernodet, G. L. Challis, *Microbiology (Reading)* **2007**, *153* (Pt 5), 1405.

[2] M. Izumikawa, P. R. Shipley, J. N. Hopke, T. O'Hare, L. Xiang, J. P. Noel, B. S. Moore, *J. Ind. Microbiol. Biotechnol.* **2003**, *30* (8), 510.

[3] S. Kol, M. E. Merlo, R. A. Scheltema, M. de Vries, R. J. Vonk, N. A. Kikkert, L. Dijkhuizen, R. Breitling, E. Takano, *Appl. Environ. Microbiol.* **2010**, *76* (8), 2574.

[4] V. H. Tierrafría, H. E. Ramos-Aboites, G. Gosset, F. Barona-Gómez, *Microb. Biotechnol.* **2011**, *4* (2), 275.

[5] Z. Hojati, C. Milne, B. Harvey, L. Gordon, M. Borg, F. Flett, B. Wilkinson, P. J. Sidebottom, B. A. Rudd, M. A. Hayes, C. P. Smith, J. Micklefield, *Chem. Biol.* **2002**, *9* (11), 1175.

[6] T. Taguchi, K. Itou, Y. Ebizuka, F. Malpartida, D. A. Hopwood, C. M. Surti, K. I. Booker-Milburn, G. R. Stephenson, K. Ichinose, *J. Antibiot. (Tokyo)* **2000**, *53* (2), 144.

[7] X. Lin, R. Hopson, D. E. Cane, *J. Am. Chem. Soc.* **2006**, *128* (18), 6022.

[8] Y. Sasaki, H. Oguchi, T. Kobayashi, S. Kusama, R. Sugiura, K. Moriya, T. Hirata, Y. Yukioka, N. Takaya, S. Yajima, S. Ito, K. Okada, K. Ohsawa, H. Ikeda, H. Takano, K. Ueda, H. Shoun, *Sci. Rep.* **2016**, *6*, 22038.

[9] B. Gust, G. L. Challis, K. Fowler, T. Kieser, K. F. Chater, *Proc. Natl. Acad. Sci. USA* **2003**, *100* (4), 1541.

[10] M. Biarnes-Carrera, C. K. Lee, T. Nihira, R. Breitling, E. Takano, *ACS Synth. Biol.* **2018**, *7* (4), 1043.

[11] T. J. O'Connor, P. Kanellis, J. R. Nodwell, *Mol. Microbiol.* **2002**, *45* (1), 45.

[12] G. L. Challis, *Microbiology (Reading)* **2008**, *154* (Pt 6), 1555.

[13] N. Funa, M. Funabashi, E. Yoshimura, S. Horinouchi, *J. Biol. Chem.* **2005**, *280* (15), 14514.

[14] F. Barona-Gómez, S. Lautru, F. X. Francou, P. Leblond, J. L. Pernodet, G. L. Challis, *Microbiology (Reading)* **2006**, *152* (Pt 11), 3355.

[15] F. Kopp, U. Linne, M. Oberthur, M. A. Marahiel, *J. Am. Chem. Soc.* **2008**, *130* (8), 2656.

[16] Y. Tong, P. Charusanti, L. Zhang, T. Weber, S. Y. Lee, *ACS Synth. Biol.* **2015**, *4* (9), 1020.

[17] B. Zhao, X. Lin, L. Lei, D. C. Lamb, S. L. Kelly, M. R. Waterman, D. E. Cane, *J. Biol. Chem.* **2008**, *283* (13), 8183.

[18] J. S. Feitelson, D. A. Hopwood, *Mol. Gen. Genet.* **1983**, *190* (3), 394.

[19] B. Bednarz, A. Millan-Oropeza, M. Kotowska, M. Świat, J. J. Quispe Haro, C. Henry, K. Pawlik, *Front. Microbiol.* **2021**, *12*, 616050.

[20] H. Ma, K. Kendall, *J. Bacteriol.* **1994**, *176* (12), 3800.

[21] a) J. H. Lee, J. S. Yoo, Y. Kim, J. S. Kim, E. J. Lee, J. H. Roe, *mBio* **2020**, *11* (2); b) A. Zorro-Aranda, J. M. Escorcia-Rodríguez, J. K. González-Kise, J. A. Freyre-González, *Sci. Rep.* **2022**, *12* (1), 2840.

[22] T. L. Bailey, J. Johnson, C. E. Grant, W. S. Noble, *Nucleic Acids Res.* **2015**, *43* (W1), W39.

[23] Y. Jeong, J. N. Kim, M. W. Kim, G. Bucca, S. Cho, Y. J. Yoon, B. G. Kim, J. H. Roe, S. C. Kim, C. P. Smith, B. K. Cho, *Nat. Commun.* **2016**, *7*, 11605.
